# Machine learning prediction model based on enhanced bat algorithm and support vector machine for slow employment prediction

**DOI:** 10.1371/journal.pone.0294114

**Published:** 2023-11-09

**Authors:** Yan Wei, Xili Rao, Yinjun Fu, Li Song, Huiling Chen, Junhong Li

**Affiliations:** 1 Department of Information Technology, Wenzhou Vocational College of Science and Technology, Wenzhou, 325006, China; 2 The Section of Employment, Wenzhou Vocational College of Science and Technology, Wenzhou, 325006, China; 3 Department of Computer Science and Artificial Intelligence, Wenzhou University, Wenzhou, 325035, China; 4 School of Public Health and Management, Wenzhou Medical University, Wenzhou, 325035, China; UNITEN: Universiti Tenaga Nasional, MALAYSIA

## Abstract

The employment of college students is an important issue that affects national development and social stability. In recent years, the increase in the number of graduates, the pressure of employment, and the epidemic have made the phenomenon of ’slow employment’ increasingly prominent, becoming an urgent problem to be solved. Data mining and machine learning methods are used to analyze and predict the employment prospects for graduates and provide effective employment guidance and services for universities, governments, and graduates. It is a feasible solution to alleviate the problem of ’slow employment’ of graduates. Therefore, this study proposed a feature selection prediction model (bGEBA-SVM) based on an improved bat algorithm and support vector machine by extracting 1694 college graduates from 2022 classes in Zhejiang Province. To improve the search efficiency and accuracy of the optimal feature subset, this paper proposed an enhanced bat algorithm based on the Gaussian distribution-based and elimination strategies for optimizing the feature set. The training data were input to the support vector machine for prediction. The proposed method is experimented by comparing it with peers, well-known machine learning models on the IEEE CEC2017 benchmark functions, public datasets, and graduate employment prediction dataset. The experimental results show that bGEBA-SVM can obtain higher prediction Accuracy, which can reach 93.86%. In addition, further education, student leader experience, family situation, career planning, and employment structure are more relevant characteristics that affect employment outcomes. In summary, bGEBA-SVM can be regarded as an employment prediction model with strong performance and high interpretability.

## 1 Introduction

Employment is the most basic livelihood, and the employment of college students is an important issue related to people’s livelihood and the stable development of society. In recent years, China’s economic development has entered a new normalized stage, and the number of graduates as well as the increase in employment pressure. It has led to more college graduates who do not intend to be employed immediately after completing their studies, and thus actively or passively become a ’slow employment’ group. The phenomenon of ’slow employment’ has become a prominent problem in the employment of college graduates, in addition to the impact brought about by the epidemic, the phenomenon of ’slow employment’ of college students has become more prominent. It brings a greater negative impact on China’s economic development, social stability, and talent training in many aspects.

Understanding the basic situation of the ’slow employment’ group of college students, analyzing the causes of the ’slow employment’ phenomenon, and digging out the key influencing factors, provides a set of scientific and effective solutions to prevent the ’slow employment’ behavior and crack the ’slow employment’ phenomenon. This is of great significance to promote higher quality and fuller employment of graduates. The employment landscape for college students and graduates has become more challenging due to the COVID-19 pandemic [1]. Shi [2] pointed out that the record-breaking number of graduates and the problem of career decision-making for graduates due to the economic recession have become obstacles to the active employment of college students. Researchers and college educators are increasingly concerned about graduate employment and the potential problems that may exist in the future career choices of current students. Li and Zhang [3] constructed a decision tree model based on big data to provide effective help for college students to solve the problem of slow employment in terms of scientific decision-making, precise guidance, accurate service, and time-sensitive assistance. Wang and Li [4] verified the mechanism of the influence of employment value on the willingness to choose slow employment by means of a survey document for students of several universities in Haidian and Changping districts of Beijing. The study found that long-term post-employment income and employment costs have a facilitating effect on positive ’slow employment’ choices, employment anxiety only plays a mediating role, and short-term income plays a negative role for ’slow employment’. This study contributes to a deeper understanding of college students’ career choices and provides a reference for full employment.

At present, data mining technology has been applied in the fields of academic early warning [5], career planning [6], and teaching assessment [7]. The database of student employment information also contains a large number of valuable laws. With the development of computer technology, some machine learning methods [8] or hybrid models [9,10] have gradually come into the public’s view and become an important method to solve the prediction problem. Of course, these techniques have also received attention in the field of education. Rahman et al [11] used data mining techniques for feature selection and predicted graduate employment using techniques such as K-Nearest Neighbor, Naive Bayes, Decision Trees, Neural Networks, Logistic Regression and Support Vector Machines and then analyzed the data based on Rapid Miner. Chen et al [12] used the road factor score approach to systematically analyze and assess the comprehensive employability of graduates to scientifically guide students in their search for suitable careers. Bharambe et al [13] used data mining techniques to assess the employability of students by using a classification model to intelligently predict which types of companies’ needs the skills acquired by students are suitable for. Zhao et al [14] proposed a random forest algorithm to select features for the employee retention rate of ’double-class’ university graduates, and based on the principal component analysis, it was found that the economic levels factors such as regional gross domestic product, wages, the average sales price of commercial properties, and the unemployment rate were the main factors affecting employment mobility in each province and city, and then a back propagation neural network was used to predict and obtain high accuracy and stability. Zhao et al [15] proposed a model for predicting the employment situation of college graduates based on long- and short-term memory recurrent neural networks. The model can effectively reflect the complex characteristics of university graduate employment data and the nonlinear dynamic interaction of influencing factors, and the data that mainly affect the employment situation were selected for prediction. It is compared with the traditional statistical method based on the cluster analysis technique, and the results show that the technique has higher prediction accuracy and reliability. Tu et al [16] proposed a model for predicting the entrepreneurial intentions of graduate students based on a chaotic local search sine cosine algorithm, random forest, and support vector machine, and demonstrated the importance of components such as major, gender, general student type, grade point average and total credits in influencing the choice of entrepreneurial intentions. Gao et al [17] proposed an intelligent prediction model of employment stability with a multi-group slime mould algorithm combined with support vector machines, which showed better prediction results and demonstrated the association of current employment monthly salary, first employment monthly salary, change of employment location, degree of first employment major affiliation, and salary difference on students’ employment stability.

To predict and assess the ’slow employment’ phenomenon of college students, this work presented and used bGEBA-SVM, a wrapper feature selection approach based on the improved bat algorithm (GEBA) and support vector machine. First, to enhance the optimization capability of the feature subset search method, Gaussian distribution-based strategy and Elimination Strategy were introduced into the bat algorithm to enhance the global optimization capability of the algorithm and to improve the population quality.

In recent years, many excellent optimization methods have been successively proposed, which include colony predation algorithm (CPA) [18], Harris hawks optimization (HHO) [19], slime mould algorithm (SMA) [20,21], bat algorithm (BA) [22], firefly algorithm (FA) [23], sine cosine algorithm (SCA) [24], butterfly optimization algorithm (BOA) [25], ant colony algorithm for the continuous domain (ACOR) [26], Runge Kutta optimizer (RUN) [27], rime optimization algorithm (RIME) [28], weighted mean of vectors (INFO) [29], and hunger games search (HGS) [30], chaotic whale optimizer (CWOAII) [31], hybridizing sine cosine algorithm with differential evolution (SCADE) [32], chaotic bat algorithm (CBA) [33], a bat algorithm based on collaborative and dynamic opposition-based learning (CDLOBA) [34], hybrid bat algorithm (RCBA) [35]. To test optimization performance of the proposed GEBA, it is compared with 10 advanced peers on 30 benchmark functions from IEEE CEC2017 [36]. The experimental results are then analyzed by Wilcoxon’s Signed-Rank Test (WSRT) [35] and Friedman test (FT) [37] to finally verify its excellent optimization performance. Second, the experimental results demonstrate the better interpretability and predictive performance of bGEBA-SVM by comparing it with five similar methods and four advanced prediction models through public datasets and graduate employment prediction datasets from the class of 2022 college graduates in Zhejiang Province.

The remaining structure of the paper is set up as follows. Section 2 presents the graduate employment prediction dataset and the proposed bGEBA-SVM prediction method. Section 3 implements a benchmark function experiment based on IEEE CEC2017 to validate GEBA’s optimization capacity. Section 4 validates the prediction ability of bGEBA-SVM with the public dataset and the graduate employment prediction dataset. Section 5 discusses the suggested approach and the experimental results in further detail. Section 6 summarizes this study and proposes goals for future research based on the existing foundation.

## 2 Materials and methods

### 2.1 Graduate employment prediction dataset

In this study, 1,694 graduates of the class of 2022 from Zhejiang universities were selected for the study, and predictions were made based on 18 characteristics. The details of the Graduate Employment Prediction (GEP) dataset are shown in Table 1.

**Table 1:** Details of the GEP dataset.

No.	Features	Remark
**A1**	Gender	Divided into male and female, divided into expressed by 1 and 2.
**A2**	Employment structure	According to the students’ majors, they are divided into science and technology, economic and management, agriculture and other categories, which are indicated by 1,2,3,4.
**A3**	Student Category	Urban and rural students are divided into 1 and 2.
**A4**	Student Leadership Experience	Whether the student has served as a student leader during college, yes is indicated by 1, no is indicated by 2.
**A5**	Academic Achievement	Academic performance is mainly judged by whether students have won scholarships during their school years, indicated by 1 and 2.
**A6**	Professional Interest	Students’ interest in their majors is classified as very uninterested, less interested, interested, more interested, and very interested, respectively, indicated by 1, 2, 3, 4, and 5.
**A7**	Professional recognition	According to the students’ recognition of their majors, 1,2,3 are used for bad, moderate and good respectively.
**A8**	Employment mentality	According to students’ employment mentality, 1,2,3 are used for bad, average, and good respectively.
**A9**	Career orientation	According to students’ career orientation, self-worth, personal interests, salary, working environment, employment prospect, professional alignment and job stability are indicated by 1,2,3,4,5,6 and 7 respectively.
**A10**	Career Planning	Whether students have a clear career plan, indicated by 1 and 2.
**A11**	Preparation for school	Whether the student has a plan to prepare for school, indicated by 1 and 2.
**A12**	Family Situation	Students’ family financial situation is classified as poor, moderate or good, indicated by 1,2,3.
**A13**	Family parenting style	According to the authoritative, permissive and democratic parenting style of the family, 1,2,3 are used respectively.
**A14**	Parental attitude	Parents’ attitudes toward students’ slow employment were categorized by 1,2,3 according to their support, opposition, and indifference, respectively.
**A15**	Professional employment situation	1,2,3 according to the serious, average and optimistic situation of their majors.
**A16**	Peer influence	According to whether they are influenced by peer employment, yes is indicated by 1, no is indicated by 2.
**A17**	Network employment atmosphere	According to the influence of the network employment atmosphere on personal job search, the influence is indicated by 1, and the absence of influence is indicated by 2.
**A18**	Employment guidance strength	1,2,3 for weak, moderate and strong career guidance in colleges and universities respectively.

Since the above-mentioned data did not involve ethical issues, the review committee/ethics committee of Wenzhou Vocational College of Science and Technology granted an exemption from ethical review.

### 2.2 Bat algorithm

The bat algorithm mainly simulates the behavior of bats to find tiny insects foraging through an echolocation system. In the bat algorithm, each bat (search agent) flies at a random velocity *v*_*i*_ at the location *x*_*i*_ (solution of the problem) while the bats have different wavelengths, loudness *A*_*i*_, and pulse emissivity *r*. When a bat finds prey, its frequency, loudness, and pulse emissivity change for the best solution selection. The specific, more detailed procedure of the bat algorithm is as follows.

First, each bat generates ultrasonic frequencies *f*_*i*_ according to random, as shown in Eq (1).

$$f_{i}=f_{min}+(f_{max}-f_{min})\times \beta$$ (1)

where *β* is a 0 to 1 random vector, and *f*_*min*_ and *f*_*max*_ are the minimum and maximum values of *f*_*i*_ which are set to 0 and 2, respectively.

Then, each bat updates its velocity $v_{i}^{t}$ according to its current velocity $v_{i}^{t-1}$ and the distance between its current position $x_{i}^{t-1}$ and the optimal position *x*_*best*_ further updates the bat’s current position $x_{i}^{t}$ according to $v_{i}^{t}$. Eqs (2) and (3) are used to calculate $v_{i}^{t}$ and $x_{i}^{t}$, respectively.


$$v_{i}^{t}=v_{i}^{t-1}+(x_{i}^{t-1}-x_{best})\times f_{i}$$ (2)

$$x_{i}^{t}=x_{i}^{t-1}+v_{i}^{t}$$ (3)

When the global optimal solution is updated, each local solution *x*_*old*_ in the current population is updated, as shown in Eq (4).

$$x_{new}=x_{i}^{t}+\varepsilon \times A^{t}$$ (4)

where *ε* denotes a random number obeying a uniform distribution between -1 and 1, and *A*^*t*^ denotes the average loudness of all bats.

After the position is updated, the bat makes a greedy choice between the current position *x*_*old*_ and the updated position *x*_*new*_. When the bat position is updated, the loudness *A* and pulse frequency *r* are updated, as shown in Eqs (5) and (6).

$$A^{t+1}=\alpha^{t}\times A^{t}$$ (5)

$$r^{t+1}=r^{t}(1-e^{-\gamma t})$$ (6)

where *α* and *γ* are constants set to 0.9, and *r* and *A* are set to 0.5.

The pseudo-code of the bat algorithm is shown in Algorithm 1.

**Algorithm 1. Pseudocode for BA**.

**Initialize** the algorithm parameters: *f*_*min*_, *f*_*max*_, *α*, *γ*, r, A

**Initialize** the population

Evaluate the fitness value of each search agent

*FEs* = *FEs*+*N*

**While** *FEs*≤MaxFEs

**For** *i* = 1:*N*

Update the frequency *f*_*i*_ of $x_{i}^{t}$ by Eq (1)

Update the velocity $v_{i}^{t}$ of $x_{i}^{t}$ by Eq (2)

Update $x_{i}^{t}$ by Eq (3)

Evaluate the fitness value *fitness*_*i*_ of $x_{i}^{t}$

**If** (*rand*<*A*_*i*_)&&(*fitness*_*i*_<*fitness*_*best*_)

Calculate the new position *x*_*new*_ by Eq (4)

Evaluate the fitness value *fitness*_*new*_ of *x*_*new*_ and greedy selection

*FEs* = *FEs*+1

Update *A*^*t*^ by Eq (5)

Update *r*^*t*^ by Eq (6)

**End if**

**End for**

*FEs* = *FEs*+*N*

*t* = *t*+1

**End while loop**

**Return** the best solution

### 2.2 Gaussian distribution-based strategy

To obtain better optimization results, Gaussian distribution is used to optimize the way the original BA is updated, enhancing the ability of the algorithm to search for the global optimal solution in the search space. The Gaussian distribution-based strategy is calculated from Eq (7).

$$x_{new}=\left\{ \begin{array}{ll} x_{best}+A^{t}\times rand, & {Ra}_{1}\geq 0.5\\ {Gaussian(\mu }_{i},\sigma_{i}), & {Ra}_{1}<0.5 \end{array}\right.$$ (7)

where *Gaussian*(*μ*_*i*_, *σ*_*i*_) denotes the Gaussian kernel function, which obeys normal distribution. *μ*_*i*_ and *σ*_*i*_ denote the mean and standard deviation, respectively, as shown in Eqs (8) and (9).


$$\mu_{i}=\frac{x_{best}+x_{i}^{t}}{2}$$ (8)

$$\sigma_{i}=|x_{best}-x_{i}^{t}|$$ (9)

### 2.3 Elimination strategy

In this study, the elimination strategy is introduced into BA to improve the optimization capability of the algorithm. Replacing the poorer search agents in the population with the derived search agents based on the optimal solution *x*_*best*_ and the suboptimal solution *x*_*sub*_ can improve the population quality, prevent the search agents from overexploiting near the poor solution, and thus improve the algorithm accuracy. The mathematical model of the elimination strategy is as follows.

First, the individual information of the optimal solution *x*_*best*_ and the suboptimal solution *x*_*sub*_ is used to generate a new reference search agent x_ref, as shown in Eq (10).


$$x_{ref}=\frac{x_{best}+x_{sub}}{2}$$ (10)

Then, the worst 5% of individuals in the population were updated according to Eqs (11) and (12).

$$x_{i}^{t}=\left\{ \begin{array}{ll} x_{ref}+{Ra}_{2}\times Dis, & k\geq 0.5\\ x_{ref}\times k, & k<0.5 \end{array}\right.$$ (11)

$$Dis=\sqrt{∥x_{best}-x_{sub}∥}$$ (12)

where *k* and *Ra*_2_ are random numbers between 0 and 1 that obey a uniform distribution.

### 2.4 Implementation of GEBA

To improve the optimization performance of the original BA, the Gaussian distribution-based strategy and elimination strategy were introduced in this study. Wherein, the Gaussian distribution-based strategy is used to improve the convergence accuracy of the algorithm, so Eq (4) in the original BA was replaced by Eqs (7) to (9). In the early stages of optimization, a more diverse population can speed up the algorithm’s convergence and enhance the likelihood that the algorithm will find the best solution. However, when the optimization is late a larger population diversity may cause the algorithm to waste more resources on computing inferior solutions, which is not conducive to obtaining high-quality solutions. Thus, the pseudo-code of GEBA shows that after all search agents in BA were updated by the elimination strategy, the inferior solutions in the population were replaced with new search agents in the optimal solution region, which promotes a balance between global exploration and local exploitation of the algorithm. The pseudo-code for GEBA is shown in Algorithm 2.

**Algorithm 2. Pseudocode for GEBA.**

**Initialize** the algorithm parameters: *f*_*min*_, *f*_*max*_, *α*, *γ*, r, A,

**Initialize** the population

Evaluate the fitness value of each search agent

*FEs* = *FEs*+*N*

**While** *FEs*≤MaxFEs

**For** *i* = 1:*N*

Update the frequency *f*_*i*_ of $x_{i}^{t}$ by Eq (1)

Update the velocity $v_{i}^{t}$ of $x_{i}^{t}$ by Eq (2)

Update $x_{i}^{t}$ by Eq (3)

Evaluate the fitness value *fitness*_*i*_ of $x_{i}^{t}$

**If** (*rand*<*A*_*i*_)&&(*fitness*_*i*_<*fitness*_*best*_)

Update *μ*_*i*_ by Eq (8)

Update *σ*_*i*_ by Eq (9)

Calculate the new position *x*_*new*_ by Eq (7)

Evaluate the fitness value *fitness*_*new*_ of *x*_*new*_ and greedy selection

*FEs* = *FEs*+1

Update *A*^*t*^ by Eq (5)

Update *r*^*t*^ by Eq (6)

**End if**

**End for**

**For** *i* = 1:*N*

Update *x*_*ref*_ according to *x*_*best*_ and *x*_*sub*_ by Eq (10)

Update $x_{i}^{t}$ by Eqs (11) to (12)

Evaluate the fitness value *fitness*_*i*_ of $x_{i}^{t}$ and greedy selection

**End for**

*FEs* = *FEs*+2×*N*

*t* = *t*+1

**End while loop**

**Return** the best solution

### 2.5 Proposed prediction model bGEBA-SVM

The set of optimized features may be thought of as a discrete optimization problem for feature selection, where a value of ’1’ indicates that the feature has been chosen and a value of ’0’ indicates that it has not. As a result, GEBA is further modified by discretization to create bGEBA, a variant that may be used to optimize the discrete space. The S-shaped function was selected as the GEBA transformation function in this study.

The S3 function in the S-shaped is applied to the *j*-th component of the *i*-th search agent, *x*_*i*,*j*_. If the random number between 0 and 1 is smaller than the output value *s*, the component information *xb*_*i*,*j*_ of this search agent in the discrete space is output as 1, otherwise it is output as 0.

The *j*-th component of the *i*-th search agent *x*_*i*,*j*_ is input into the S3 function in the S-shaped, and if the random number between 0 and 1 is less than the output value *s* then the output of the component information *xb*_*i*,*j*_ of this search agent in the discrete space is 1, and vice versa is 0. Eqs (13) and (14) show the calculation.


$$s=\frac{1}{1+e^{\left(-\frac{x_{i,j}}{2}\right)}}$$ (13)

$${xb}_{i,j}=\left\{ \begin{array}{cc} 1, & rand<s\\ 0, & rand\geq s \end{array}\right.$$ (14)

**Fig 1:**
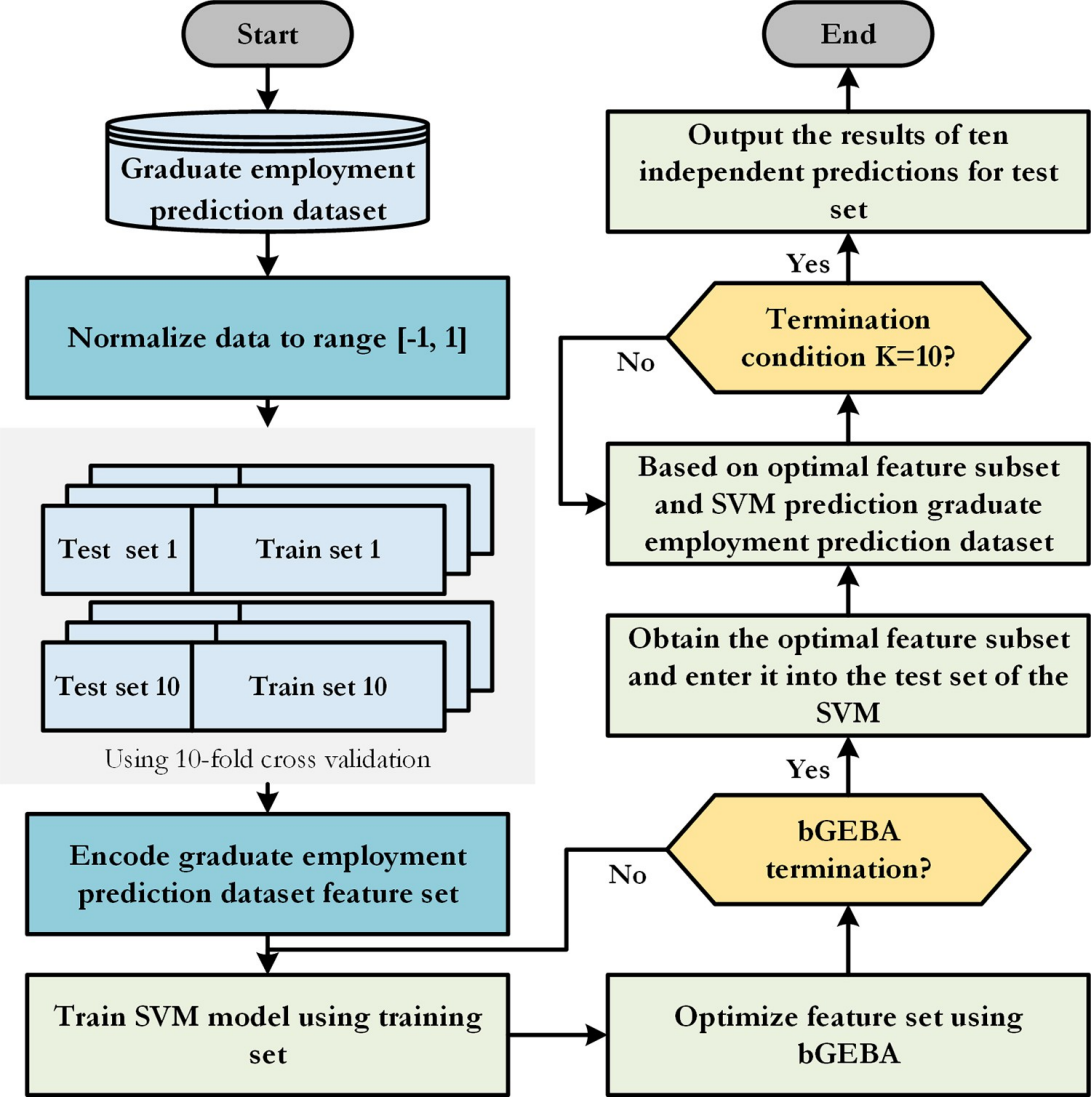


Support vector machine (SVM) [38] has excellent generalization performance as well as good performance for nonlinear and nonconvex problems, so SVM was chosen as the classifier for the GEP dataset in this study. Furthermore, a wrapper feature selection method based on the combination of bGEBA and SVM is proposed, which is called bGEBA-SVM. The prediction process of bGEBA-SVM for the GEP dataset is shown in Fig 1 (The code is available at https://github.com/Forproject1111/bGEBA-SVM).

This approach makes use of bGEBA’s exceptional ability to search for the best option to optimize the feature subset. The feature subset is fed into the SVM as input for training, and the SVM’s prediction output serves as the evaluation criterion (fitness function) for this collection of feature subsets. The fitness function *fitness* is generated using Eq (15). The process is repeatedly iterated until the feature subset that gives the classifier the best performance is attained.

$$fitness=\omega_{1}\times (1-Accuracy)+\omega_{2}\times \frac{R}{D}$$ (15)

where, *ω*_1_ and *ω*_2_ make two weight parameters that are used to measure the impact of the model prediction accuracy and the number of selected features on the feature subset evaluation. Since the prediction accuracy of the model is the focus of the study, *ω*_1_ and *ω*_2_ are set to 0.99 and 0.01, respectively.

where *ω*_1_ and *ω*_2_ are two weight factors that are used to assess how well the model predicts the future and how many features were chosen for the feature subset analysis. Since the study’s main focus is the model’s prediction accuracy, *ω*_1_ and *ω*_2_ are set to 0.99 and 0.01, respectively.

## 3 Experiments on benchmark functions

In the prediction method based on the wrapper feature selection, swarm intelligence optimization algorithms are used as a key part of them to optimize the subset of features trained by the input model. Therefore, the optimization capability of the swarm intelligence optimization algorithm is one of the important factors affecting the prediction results. To explore the optimization performance of GEBA, this section sets up the benchmark function experiments for validation.

### 3.1 Experiment setup

The algorithm’s search way can be divided into global exploration and local exploitation. The global exploration capability indicates the algorithm’s ability to search for optimal solutions in unknown regions of the search space, increasing the probability of the algorithm avoiding local extremes. However, the probability of obtaining a poor solution is likewise elevated, which is not conducive to improving the accuracy of the current optimal solution. The local exploitation capability indicates the ability of the algorithm to further exploit near the current solution and improve the quality of the optimal solution. But this may make the algorithm fall into a local optimum dilemma. When the global exploration ability and local exploitation ability of the algorithm are balanced, the optimization ability of the algorithm can be fully utilized, and thus better optimization results can be obtained. To more comprehensively verify the optimization performance of the algorithm, this section verifies the optimization capability of the algorithm based on the IEEE CEC2017 benchmark function [36], and the details of IEEE CEC2017 are shown in Table 2. In addition, to ensure the fairness of the experimental results, the public parameters of the benchmark function experiments are set uniformly, and the public parameters as well as the experimental environment are shown in Table 3.

**Table 2:** Details of the 30 benchmark functions for IEEE CEC2017.

Class.	Function
**Unimodal function**	F1 ~ F3
**Multimodal function**	F4 ~ F10
**Hybrid function**	F11 ~ F20
**Composition function**	F21 ~ F30

**Table 3:** Parameter settings for the benchmark function experiments.

	Name	Remark	Value
**Public parameters**	*MaxFEs*	Maximum number of function evaluations	300,000
	*N*	Maximum number of search agents	30
	*Dim*	Maximum dimension of the objective function	30
	*Run*	Number of independent program runs	30
	*Ub*	The maximum value in the search space	100
	*Lb*	The minimum value in the search space	-100
**Experimental environment**	CPU	11th Gen Intel(R) Core(TM) i5-11400H	
	GPU	NVIDIA GeForce RTX 3050	
	RAM	16GB	
	Operating System	Windows 10 Professional	
	Software	MATLAB R2018b	

### 3.2 Ablation experiment

To verify the significance of the Gaussian distribution-based strategy and elimination strategy for the performance improvement of the algorithm, this subsection set up the ablation experiments of the optimization strategies. The two optimization strategies were introduced into BA separately, and the details of the ablation experiment comparison method are shown in Table 4. The comparison was carried out by ranking in both WSRT and FT nonparametric tests as well as convergence tests.

**Table 4:** Ablation experiment comparison method setup.

Algorithm	Strategy I (Gaussian distribution-based strategy)	Strategy II (Elimination strategy)
**GEBA**	1	1
**GBA**	1	0
**EBA**	0	1
**BA**	0	0

**Note:** ’1’ indicates that the strategy is selected, ’0’ indicates that the strategy is not selected.

**Fig 2:**
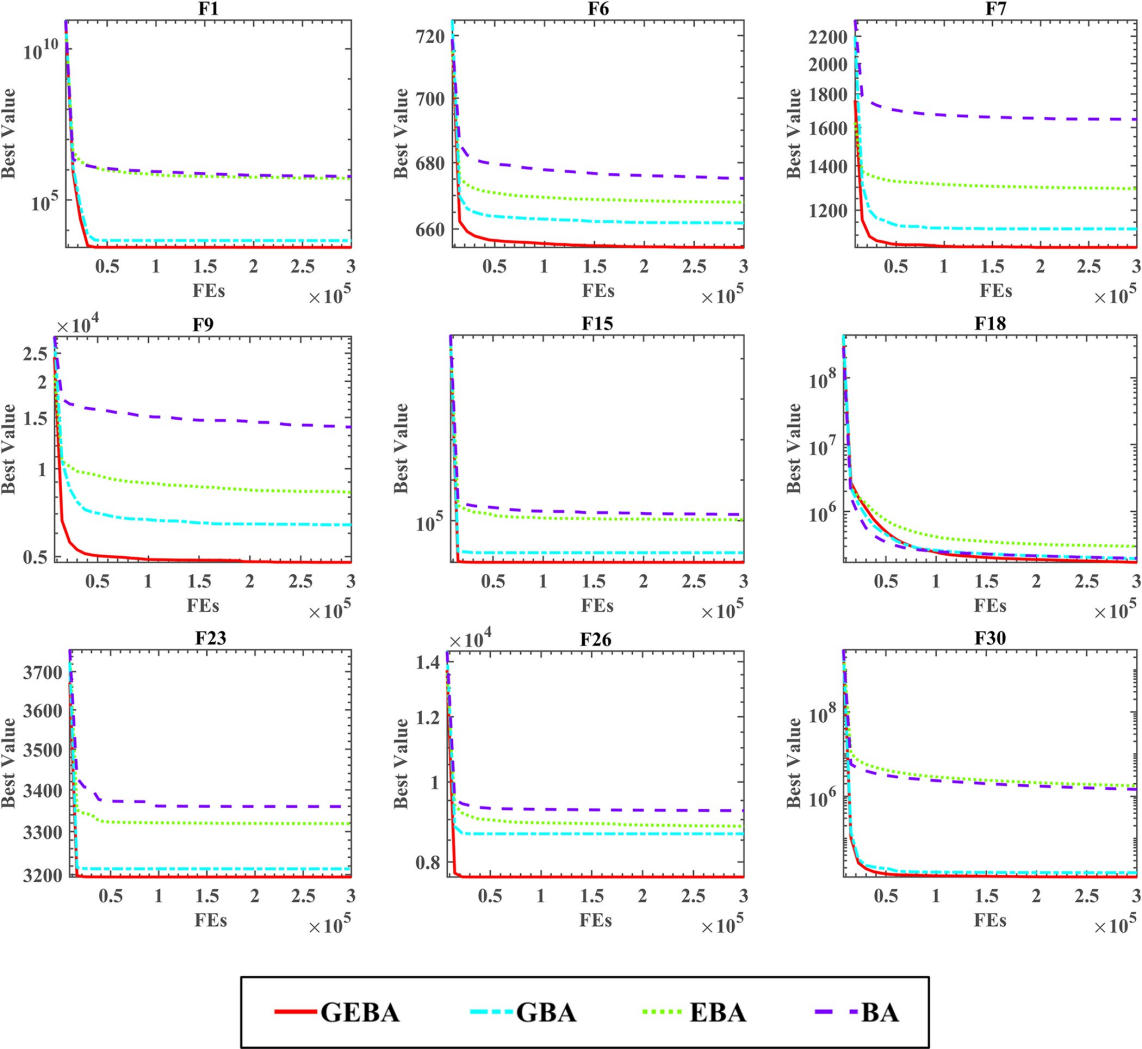


Table 5 shows the comparison results and rankings of the four methods, from the table it can be seen that the average rankings of GEBA for WSRT and FT are 1.37 and 1.88 respectively with the best performance, followed by GBA. The results of the two test rankings of EBA and BA are different, EBA performs better in the WSRT ranking, while the introduction of the elimination strategy in the FT ranking instead reduces the optimization performance of the algorithm. However, the comparison between GEBA and GBA shows that the performance improvement of the GBA algorithm is more obvious by the elimination strategy. Fig 2 shows the convergence of the four methods, and it is clear from the figure that the convergence curve of GEBA is at the bottom of all methods, and GEBA converges faster except for the F18 function.

**Table 5:** Comparison results of ablation experiment.

Algorithm	P^+^/P^-^/P ^=^	WSRT		FT	
		Mean Rank	Rank	Mean Rank	Rank
**GEBA**	**N/A**	**1.37**	**1**	**1.88**	**1**
**GBA**	7/0/23	2.03	2	2.09	2
**EBA**	21/0/9	3.27	3	3.02	4
**BA**	20/0/10	3.33	4	3.01	3

**Note:** N/A indicates null value, **bold** indicates optimal result.

In summary, the experiment in this subsection demonstrates that the combination of two optimization strategies outperforms the optimization effect of individual strategies and has more significant performance improvement for algorithm optimization.

### 3.3 Search history analysis

To explore the search process of GEBA for optimal solutions to different optimization problems, this subsection was experimentally verified by performing 1-dimensional search history, 2-dimensional function top view, and average fitness value error.

**Fig 3:**
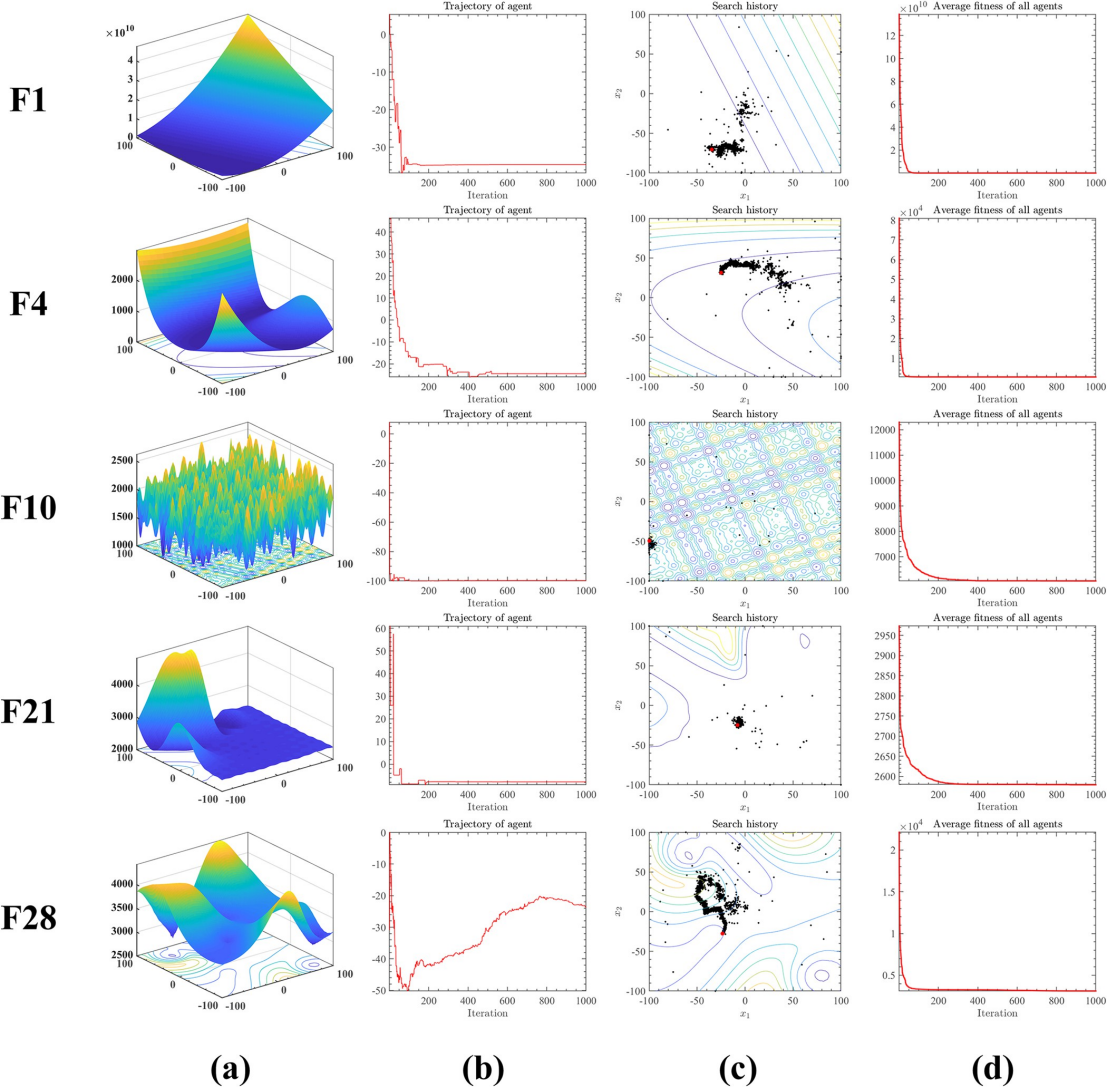


Fig 3 shows the historical search trajectory of GEBA on different optimization problems. Fig 3(A) shows the corresponding 3D image of the function. Fig 3(B) shows that the GEBA searches for larger steps in the search space in the early iterations, and in the late iterations as the global exploration ability decreases, the local search ability increases making the GEBA gradually converge to the optimal value in the dimension. From Fig 3(C), it can be seen that on the cross-section of the search space, the black dots indicate the historical locations of all search agents and the red dot indicates the location of the optimal solution. It can be seen that most of the search agents’ locations are clustered, and only a few of them are scattered in various regions of the search space. Of course, by looking at F1, F4, and F28 it is easy to see that in addition to a part of the historical positions clustering around the optimal solution, there is also a part of clustering around the suboptimal or local optimal solution. But in the end, the advantage of GEBA is to get rid of the local optimum and thus obtain a higher quality solution. Fig 3(D) shows the average fitness value curve between populations, similar to the fitness value convergence curve as the number of iterations increases the curve gradually converges to a smaller value. In addition to illustrating the gradual convergence of GEBA to the optimal solution, the average fitness value convergence curve also indicates that the differences among the search agents are smaller and more consistent with the optimization process in the later iterations.

In summary, in the optimization process, GEBA gradually converges to the optimal position based on the information provided by all search agents, the global exploration of the population gradually becomes local exploitation, and GEBA has a strong ability to jump out of the local optimal solution.

### 3.4 Stability experiment

To explore the stability of the algorithm in handling high-dimensional optimization problems, this subsection tested the optimization performance of GEBA and BA at 50 and 100 dimensions.

**Fig 4:**
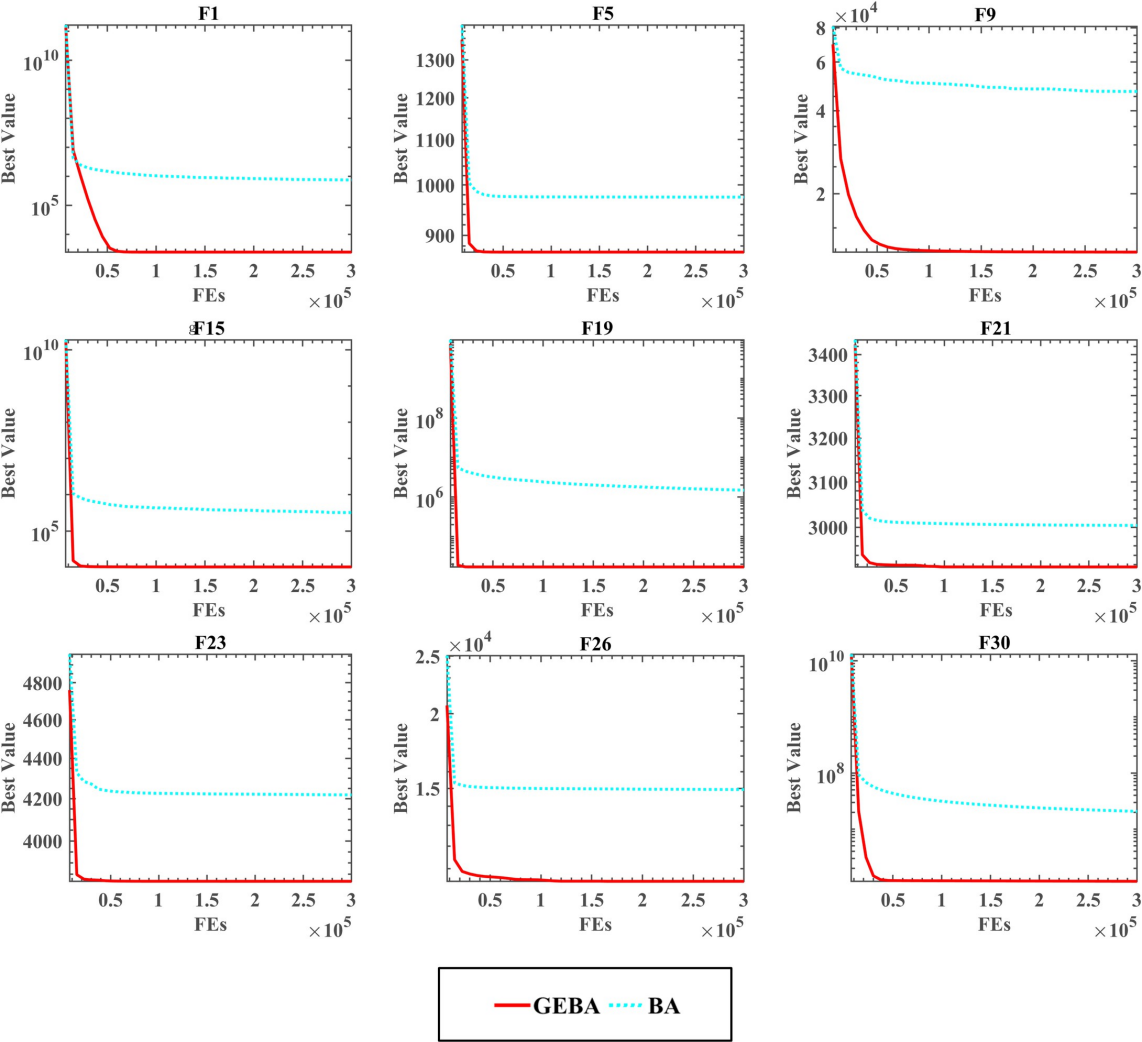


**Fig 5:**
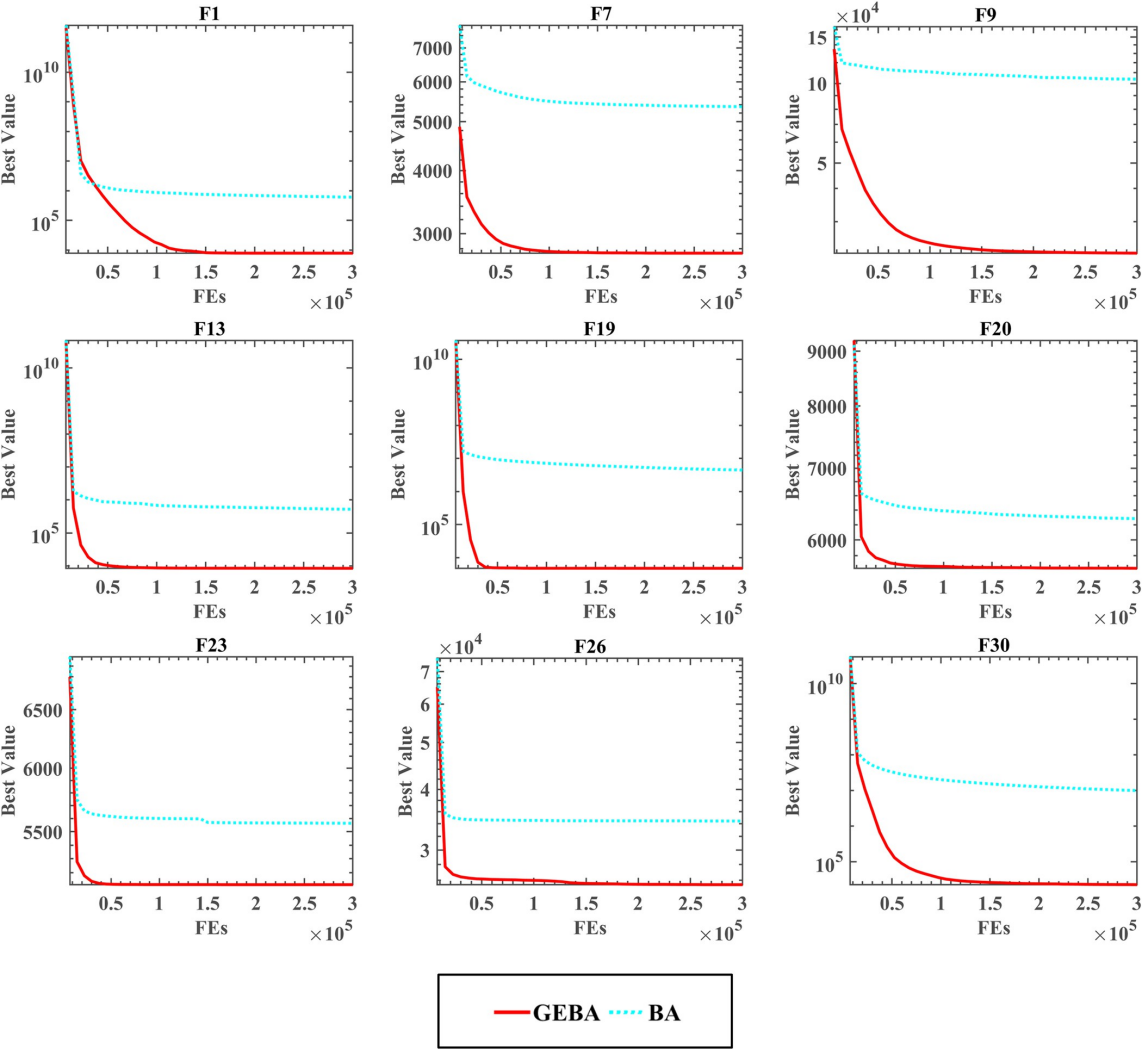


Table 6 shows the comparative results and rankings of the algorithms tested at high dimensions. GEBA outperforms BA in both dimensions with more than 19 functions, and BA performs better in only one function. There is no significant difference between the two algorithms in other functions, and the optimization performance of GEBA and BA is approximately equal. The ranking of the two statistical nonparametric tests, WSRT and FT, shows that GEBA has better optimization capability and robustness in handling different optimization problems. In addition, the convergence speed and convergence accuracy of GEBA can be seen in Figs 4 and 5, which show that GEBA performs well in high dimensions.

**Table 6:** Comparative results of scalability experiment.

Algorithm	Dim	P^+^/P^-^/P ^=^	WSRT		FT	
			Mean Rank	Rank	Mean Rank	Rank
**GEBA**	**50**	**N/A**	**1.17**	**1**	**1.25**	**1**
**BA**		22/1/7	1.83	2	1.75	2
**GEBA**	**100**	**N/A**	**1.13**	**1**	**1.23**	**1**
**BA**		19/1/10	1.87	2	1.77	2

**Note:** N/A indicates null value, bold indicates the optimal result.

Combined with the above analysis, GEBA’s optimization ability and convergence are significantly improved when dealing with high-dimensional problems.

### 3.5 Comparative experiment on GEBA with advanced peers

To objectively evaluate the algorithm optimization performance, GEBA was compared with 10 advanced similar methods, and the parameters of the 10 comparison methods were set as shown in Table 7.

**Table 7:** Details of the comparison algorithm parameters.

Algorithm	Parameter and value
**GEBA**	A=0.5,r=0.5,α=0.9,γ=0.9,p=0.45
**BA**	A=0.5,r=0.5,α=0.9,γ=0.9
**FA**	α=0.2,β=2,γ=1,δ=0.05×(ub−lb),DampingRatio=0.98
**SCA**	*a* = 2
**BOA**	*P* = 0.8,*a* = 0.1,*c* = 0.01
**ACOR**	*q* = 0.2,*ξ* = 0.6
**CWOAII**	*b* = 1,*p* = 0.5,
**SCADE**	$a=2,F=rand(0.2,0.8),P_{c}=0.8$
**CBA**	A=0.5,r=0.5,α=0.9,γ=0.9
**CDLOBA**	$A_{i}^{0}\epsilon (1,2),r_{i}^{0}\epsilon (1,2),\alpha =0.9,\gamma =0.9$
**RCBA**	A=0.7,r=0.5,α=0.9,γ=0.9,u=randn(0,1),p=rand(0,1)

**Fig 6:**
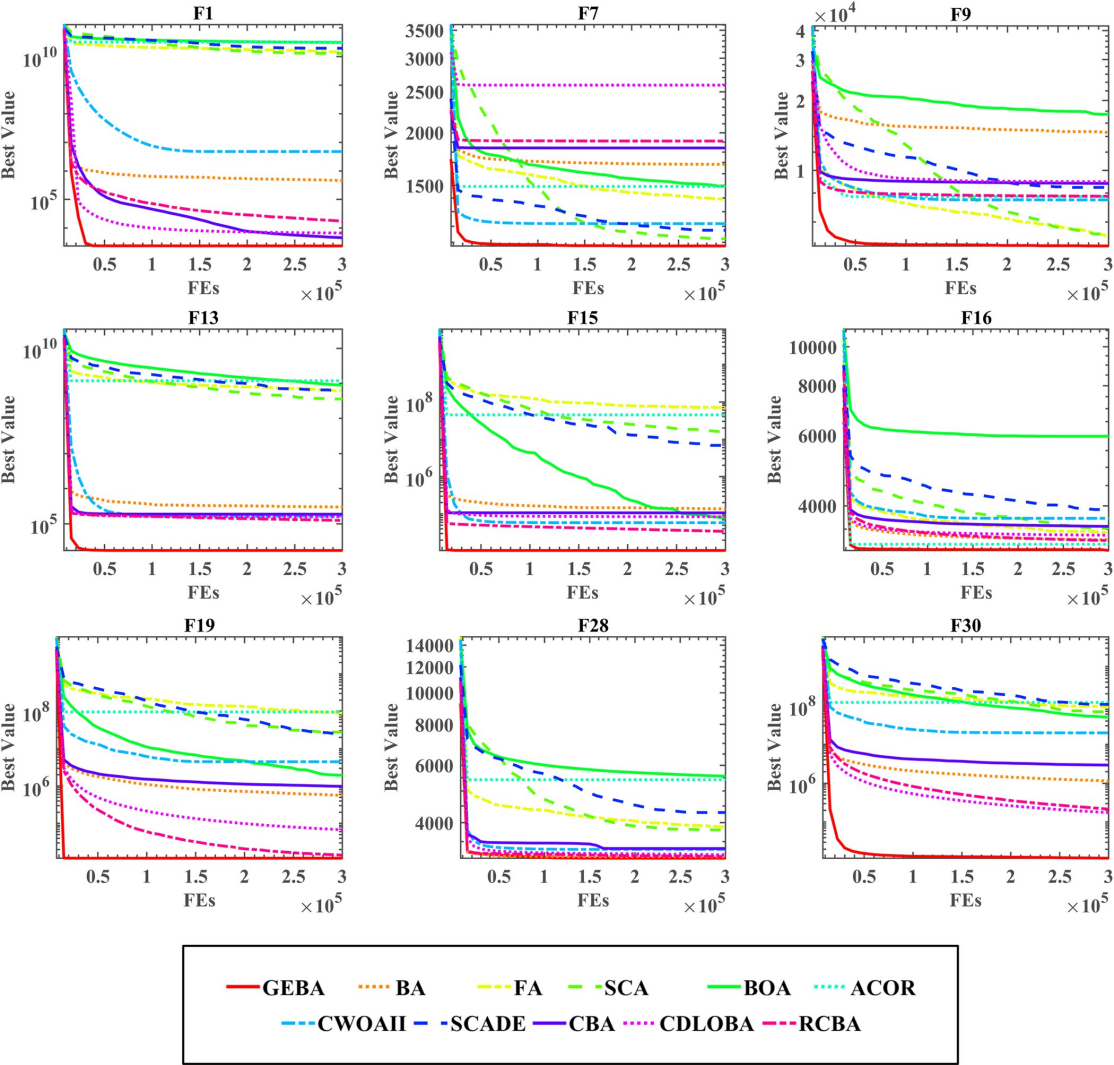


First, the performance of the 11 methods was verified by evaluating the mean and standard deviation of 30 independently run experiments on each function, and the experimental results are shown in Table 8. Observing the data in the table, it can be seen that the best performing algorithm among the 30 benchmark functions is GEBA. Among them, GEBA has 16 optimal means and 7 minimum standard deviations. Although GEBA is less stable compared to FA, the optimization performance of GEBA is still stable among all compared methods, and GEBA has a better overall optimization performance. Second, the results of the comparison between WSRT and FT statistics in Table 9 also show that GEBA ranks higher compared to the other methods. In the comparison results of P-values between GEBA and other methods, the number of P^+^ is at least 19 and the number of P^-^ is at most 6. It indicates that GEBA performs better and that these results are statistically significant. For the WSRT ranking, GEBA had the best average ranking of 2.50, followed by FA. For the FT ranking, GEBA had the best average ranking of 3.20, followed by RCBA. Third, the convergence curve images of the 11 algorithms are shown in Fig 6. Observing the images it is easy to see that at about 20,000 to 50,000 evaluations, the curve of GEBA reaches convergence and the solution quality is higher.

**Table 8:** Optimization results of GEBA with advanced peers on the IEEE CEC2017.

No.	Item		GEBA		BA		FA		SCA		BOA		ACOR	
**F1**	**Mean**		**2.3705E+03**		4.5341E+05		1.4307E+10		1.3052E+10		3.1239E+10		3.2150E+10	
	**Stdv**		2.2442E+03		2.6241E+05		1.6520E+09		2.4524E+09		8.7231E+09		1.1646E+10	
**F2**	**Mean**		**2.0000E+02**		2.0000E+02		1.1371E+34		1.2405E+34		1.7522E+53		5.9426E+46	
	**Stdv**		4.0964E-05		7.0456E-05		1.7435E+34		4.4769E+34		4.9253E+53		3.2002E+47	
**F3**	**Mean**		**3.0000E+02**		3.0011E+02		5.8967E+04		3.8247E+04		4.0168E+04		2.2633E+05	
	**Stdv**		5.5056E-04		7.7620E-02		8.2431E+03		6.4790E+03		9.1912E+03		8.3531E+04	
**F4**	**Mean**		**4.4600E+02**		4.7640E+02		1.3882E+03		1.3513E+03		9.9654E+03		5.3602E+03	
	**Stdv**		3.8399E+01		3.1673E+01		1.0929E+02		2.3941E+02		2.9812E+03		2.1037E+03	
**F5**	**Mean**		7.6734E+02		8.2207E+02		7.5832E+02		7.7795E+02		9.6354E+02		**7.5578E+02**	
	**Stdv**		4.2872E+01		8.0475E+01		1.4078E+01		2.4832E+01		4.4498E+01		5.6498E+01	
**F6**	**Mean**		6.5317E+02		6.7148E+02		**6.4394E+02**		6.5034E+02		6.9689E+02		6.5025E+02	
	**Stdv**		8.2481E+00		9.4267E+00		2.6218E+00		4.7327E+00		7.4475E+00		1.0426E+01	
**F7**	**Mean**		**1.0782E+03**		1.6828E+03		1.3920E+03		1.1219E+03		1.4916E+03		1.4914E+03	
	**Stdv**		9.1454E+01		1.7226E+02		3.8340E+01		3.0867E+01		5.8011E+01		2.2162E+02	
**F8**	**Mean**		**9.9461E+02**		1.0591E+03		1.0546E+03		1.0490E+03		1.1976E+03		1.0378E+03	
	**Stdv**		3.4460E+01		6.8890E+01		7.7087E+00		1.7773E+01		4.5640E+01		4.2136E+01	
**F9**	**Mean**		**4.7330E+03**		1.4573E+04		5.2018E+03		5.2893E+03		1.7459E+04		7.7153E+03	
	**Stdv**		8.4152E+02		4.9885E+03		5.5362E+02		9.0613E+02		2.9468E+03		2.2563E+03	
**F10**	**Mean**		**5.3306E+03**		5.8816E+03		8.0010E+03		8.0861E+03		7.3257E+03		5.8380E+03	
	**Stdv**		5.8754E+02		8.8284E+02		3.2489E+02		2.9073E+02		3.9017E+02		5.4365E+02	
**F11**	**Mean**		1.3001E+03		1.3185E+03		3.4517E+03		2.0858E+03		2.3853E+03		1.2482E+04	
	**Stdv**		5.9697E+01		6.2902E+01		5.4253E+02		2.4745E+02		6.3580E+02		1.0068E+04	
**F12**	**Mean**		**1.3063E+05**		2.6625E+06		1.4572E+09		1.1437E+09		4.2119E+09		2.8071E+09	
	**Stdv**		1.1777E+05		2.3451E+06		3.2923E+08		3.2815E+08		1.7155E+09		2.0477E+09	
**F13**	**Mean**		**1.7246E+04**		3.0066E+05		6.0712E+08		3.6010E+08		8.8515E+08		1.1901E+09	
	**Stdv**		2.1950E+04		1.2508E+05		1.7727E+08		1.2029E+08		9.4386E+08		1.3900E+09	
**F14**	**Mean**		6.5790E+03		6.4200E+03		2.2387E+05		1.4530E+05		8.3936E+03		2.5612E+06	
	**Stdv**		3.5451E+03		3.3387E+03		1.0534E+05		7.5383E+04		6.0314E+03		3.8359E+06	
**F15**	**Mean**		**1.0514E+04**		1.3754E+05		6.7609E+07		1.6075E+07		7.6201E+04		4.4835E+07	
	**Stdv**		1.1684E+04		1.0743E+05		2.6583E+07		1.3456E+07		5.2107E+04		8.8648E+07	
**F16**	**Mean**		**3.0936E+03**		3.2928E+03		3.4261E+03		3.5193E+03		5.9734E+03		3.2057E+03	
	**Stdv**		3.8304E+02		3.7744E+02		1.0848E+02		2.3734E+02		8.9726E+02		3.3761E+02	
**F17**	**Mean**		2.7039E+03		2.8681E+03		2.5252E+03		**2.3851E+03**		3.1724E+03		2.5640E+03	
	**Stdv**		3.0948E+02		3.5244E+02		1.2603E+02		1.6534E+02		3.5730E+02		7.9434E+02	
**F18**	**Mean**		2.0225E+05		1.6583E+05		3.7192E+06		2.6996E+06		1.5476E+05		1.5479E+07	
	**Stdv**		1.3513E+05		1.0290E+05		1.7244E+06		1.4790E+06		1.1742E+05		2.4967E+07	
**F19**	**Mean**		**1.1745E+04**		5.6176E+05		8.8300E+07		2.7598E+07		1.9424E+06		9.5800E+07	
	**Stdv**		1.1697E+04		1.9086E+05		3.3683E+07		1.9412E+07		4.9482E+06		2.6018E+08	
**F20**	**Mean**		2.8754E+03		3.0070E+03		**2.6144E+03**		2.6325E+03		2.8837E+03		2.7672E+03	
	**Stdv**		2.2609E+02		2.1305E+02		9.4805E+01		1.1402E+02		1.4364E+02		2.6247E+02	
**F21**	**Mean**		2.5625E+03		2.6470E+03		**2.5360E+03**		2.5555E+03		2.5589E+03		2.5520E+03	
	**Stdv**		6.5377E+01		9.1728E+01		1.1753E+01		1.9484E+01		1.8636E+02		4.5786E+01	
**F22**	**Mean**		6.7310E+03		7.1902E+03		**3.7982E+03**		8.7717E+03		8.2303E+03		6.9599E+03	
	**Stdv**		1.6000E+03		7.7308E+02		1.3136E+02		2.0167E+03		1.5286E+03		1.1147E+03	
**F23**	**Mean**		3.1750E+03		3.3127E+03		**2.9086E+03**		2.9931E+03		3.6369E+03		3.0383E+03	
	**Stdv**		1.2470E+02		1.1751E+02		1.2230E+01		3.3738E+01		1.8710E+02		8.6055E+01	
**F24**	**Mean**		3.3423E+03		3.3545E+03		**3.0638E+03**		3.1641E+03		3.3903E+03		3.1919E+03	
	**Stdv**		1.5255E+02		1.4379E+02		1.1815E+01		3.0224E+01		2.1551E+02		9.2380E+01	
**F25**	**Mean**		2.8957E+03		2.9075E+03		3.5603E+03		3.2102E+03		3.9641E+03		4.0594E+03	
	**Stdv**		1.0921E+01		2.5315E+01		9.1021E+01		1.0172E+02		2.7932E+02		6.1789E+02	
**F26**	**Mean**		7.1664E+03		8.6121E+03		**6.5244E+03**		6.8524E+03		6.5934E+03		7.9395E+03	
	**Stdv**		2.3226E+03		2.8651E+03		1.7649E+02		5.1684E+02		1.4275E+03		1.1551E+03	
**F27**	**Mean**		3.3531E+03		3.4896E+03		**3.3312E+03**		3.4047E+03		3.3818E+03		3.4508E+03	
	**Stdv**		9.9124E+01		2.6302E+02		1.5746E+01		4.1457E+01		1.2588E+02		1.2376E+02	
**F28**	**Mean**		**3.1156E+03**		3.1464E+03		3.8786E+03		3.8030E+03		5.5538E+03		5.4168E+03	
	**Stdv**		4.1269E+01		6.4123E+01		9.7264E+01		1.0474E+02		5.3604E+02		9.2144E+02	
**F29**	**Mean**		**4.4550E+03**		4.9713E+03		4.6045E+03		4.6635E+03		6.0817E+03		4.7137E+03	
	**Stdv**		3.8479E+02		4.0485E+02		2.2135E+02		2.2860E+02		6.7524E+02		6.1624E+02	
**F30**	**Mean**		**1.1756E+04**		1.1451E+06		9.0090E+07		6.7407E+07		4.9937E+07		1.2021E+08	
	**Stdv**		4.7912E+03		7.2443E+05		2.5678E+07		3.1083E+07		5.5003E+07		1.8980E+08	
**No.**			**Item**		**CWOAII**		**SCADE**		**CBA**		**CDLOBA**		**RCBA**	
**F1**			**Mean**		4.7759E+06		1.9661E+10		4.5840E+03		6.7316E+03		1.7470E+04	
			**Stdv**		2.8454E+06		2.5049E+09		5.3388E+03		6.2334E+03		4.3396E+03	
**F2**			**Mean**		1.6524E+24		8.7315E+36		1.5908E+04		7.6075E+12		2.0000E+02	
			**Stdv**		5.5291E+24		3.7337E+37		2.2922E+04		4.1550E+13		1.6292E-03	
**F3**			**Mean**		1.9798E+05		6.0784E+04		3.1715E+02		8.7311E+02		3.0104E+02	
			**Stdv**		5.5917E+04		7.2930E+03		1.0316E+01		8.4219E+02		3.4524E-01	
**F4**			**Mean**		5.7252E+02		3.4982E+03		5.0230E+02		4.7475E+02		4.8832E+02	
			**Stdv**		5.6338E+01		8.3700E+02		1.5712E+01		3.5512E+01		2.7112E+01	
**F5**			**Mean**		7.8989E+02		8.3276E+02		8.2947E+02		8.4900E+02		8.4042E+02	
			**Stdv**		5.0781E+01		1.7418E+01		7.2234E+01		6.4854E+01		7.1785E+01	
**F6**			**Mean**		6.6807E+02		6.6269E+02		6.7392E+02		6.7206E+02		6.7158E+02	
			**Stdv**		7.8560E+00		7.9340E+00		1.0730E+01		9.2247E+00		9.6409E+00	
**F7**			**Mean**		1.2176E+03		1.1749E+03		1.8403E+03		2.5920E+03		1.9105E+03	
			**Stdv**		9.4782E+01		3.8055E+01		2.3797E+02		2.8594E+02		2.4659E+02	
**F8**			**Mean**		1.0226E+03		1.0763E+03		1.0492E+03		1.1111E+03		1.0567E+03	
			**Stdv**		4.7838E+01		1.6839E+01		5.6559E+01		6.2537E+01		6.0137E+01	
**F9**			**Mean**		7.4721E+03		8.4580E+03		8.7931E+03		8.9468E+03		7.7469E+03	
			**Stdv**		1.9378E+03		1.2762E+03		2.7924E+03		1.8848E+03		2.4158E+03	
**F10**			**Mean**		6.2563E+03		8.1658E+03		5.6674E+03		5.6453E+03		5.8560E+03	
			**Stdv**		8.2243E+02		3.9791E+02		6.3158E+02		6.9304E+02		8.4839E+02	
**F11**			**Mean**		1.5640E+03		3.0285E+03		1.3283E+03		1.3052E+03		**1.2898E+03**	
			**Stdv**		1.7749E+02		4.7886E+02		8.3683E+01		8.1899E+01		5.6375E+01	
**F12**			**Mean**		4.8771E+07		2.0158E+09		1.1153E+07		3.5485E+05		2.1976E+06	
			**Stdv**		2.9124E+07		3.8957E+08		6.7945E+06		2.6009E+05		1.7573E+06	
**F13**			**Mean**		1.6477E+05		6.2654E+08		1.8648E+05		1.5652E+05		1.2405E+05	
			**Stdv**		1.1134E+05		2.1209E+08		1.3341E+05		1.0438E+05		1.0085E+05	
**F14**			**Mean**		8.2057E+05		2.8323E+05		1.3746E+04		**5.2448E+03**		8.3192E+03	
			**Stdv**		7.4654E+05		1.7832E+05		9.8286E+03		2.9953E+03		5.0199E+03	
**F15**			**Mean**		5.8413E+04		6.8435E+06		1.0676E+05		8.3862E+04		3.4126E+04	
			**Stdv**		4.7692E+04		4.0263E+06		8.7994E+04		5.1398E+04		1.9670E+04	
**F16**			**Mean**		3.7223E+03		3.9170E+03		3.5537E+03		3.3778E+03		3.2750E+03	
			**Stdv**		4.5488E+02		1.9883E+02		4.6928E+02		6.3727E+02		3.7423E+02	
**F17**			**Mean**		2.5450E+03		2.4744E+03		2.8514E+03		2.9257E+03		2.7812E+03	
			**Stdv**		2.8162E+02		1.3792E+02		3.8594E+02		2.8850E+02		3.0581E+02	
**F18**			**Mean**		3.0953E+06		4.1632E+06		1.8726E+05		**1.2040E+05**		1.6991E+05	
			**Stdv**		3.7578E+06		2.1677E+06		1.2557E+05		8.4178E+04		9.6440E+04	
**F19**			**Mean**		4.4663E+06		2.4896E+07		9.8309E+05		6.7658E+04		1.4189E+04	
			**Stdv**		3.0667E+06		1.1517E+07		5.0789E+05		2.5409E+04		1.0559E+04	
**F20**			**Mean**		2.7308E+03		2.7250E+03		2.9656E+03		2.9551E+03		3.0172E+03	
			**Stdv**		1.8520E+02		9.7889E+01		1.7050E+02		2.1766E+02		2.5666E+02	
**F21**			**Mean**		2.6044E+03		2.5804E+03		2.6382E+03		2.6104E+03		2.6471E+03	
			**Stdv**		6.7983E+01		2.1960E+01		6.8287E+01		6.5158E+01		6.1087E+01	
**F22**			**Mean**		6.8572E+03		4.5922E+03		6.7736E+03		6.8540E+03		7.3385E+03	
			**Stdv**		1.8478E+03		3.1848E+02		1.9811E+03		1.1550E+03		1.2992E+03	
**F23**			**Mean**		3.0540E+03		3.0187E+03		3.3827E+03		3.2085E+03		3.3635E+03	
			**Stdv**		8.8423E+01		3.4850E+01		1.5059E+02		1.3488E+02		1.7681E+02	
**F24**			**Mean**		3.2257E+03		3.1745E+03		3.3977E+03		3.3008E+03		3.4556E+03	
			**Stdv**		1.1649E+02		3.0082E+01		1.4055E+02		1.0618E+02		1.2799E+02	
**F25**			**Mean**		2.9534E+03		3.4679E+03		2.9071E+03		2.9288E+03		**2.8900E+03**	
			**Stdv**		3.5038E+01		1.5209E+02		2.3927E+01		3.3369E+01		9.9800E+00	
**F26**			**Mean**		7.6118E+03		7.5367E+03		9.0565E+03		1.0427E+04		9.7920E+03	
			**Stdv**		1.3179E+03		3.4718E+02		2.9138E+03		1.4718E+03		2.0138E+03	
**F27**			**Mean**		3.4078E+03		3.4407E+03		3.4950E+03		3.4404E+03		3.4145E+03	
			**Stdv**		1.2272E+02		5.0879E+01		2.1414E+02		1.0884E+02		1.0764E+02	
**F28**			**Mean**		3.3154E+03		4.3000E+03		3.3351E+03		3.1925E+03		3.1546E+03	
			**Stdv**		3.4314E+01		2.0585E+02		5.8567E+02		5.8844E+01		6.0268E+01	
**F29**			**Mean**		4.9466E+03		5.1353E+03		5.3720E+03		5.0415E+03		4.9248E+03	
			**Stdv**		4.5890E+02		2.3326E+02		4.5552E+02		4.8162E+02		4.0203E+02	
**F30**			**Mean**		1.9786E+07		1.0727E+08		2.9305E+06		1.7553E+05		2.1592E+05	
			**Stdv**		1.5200E+07		3.4359E+07		1.9924E+06		1.0041E+05		1.3892E+05	

**Note:** Bold indicates the optimal mean, and underline indicates the minimum standard deviation.

**Table 9:** Statistical test comparison results.

Algorithm	P^+^/P^-^/P ^=^	WSRT		FT	
		Mean Rank	Rank	Mean Rank	Rank
**GEBA**	**N/A**	**2.50**	**1**	**3.20**	**1**
**BA**	22/0/8	5.97	7	5.50	4
**FA**	19/6/5	5.47	2	6.08	7
**SCA**	22/4/4	5.57	4	6.11	8
**BOA**	23/0/7	8.37	11	8.27	11
**ACOR**	19/3/8	7.30	9	7.04	9
**CWOAII**	23/3/4	5.80	6	5.90	6
**SCADE**	23/5/2	7.30	9	7.42	10
**CBA**	23/0/7	6.53	8	5.86	5
**CDLOBA**	22/1/7	5.67	5	5.43	3
**RCBA**	22/1/7	5.53	3	5.17	2

**Note:** N/A indicates null value, bold indicates the optimal result.

The comprehensive analysis shows that GEBA has stronger optimization performance and faster convergence and higher convergence accuracy, so much so that it has the potential to be applied to many fields, such as image segmentation [39,40], medical diagnosis [41–43], engineering optimization [44], pulmonary hypertension diagnose [45], forecasting COVID-19 [46], spatiotemporal modeling of cardiac electrodynamics [47], automated detection of gastrointestinal diseases [48], and classification of dermatological disease [49].

## 4 Experiments on the employment prediction

### 4.1 Experiment setup

This section set up a series of experiments to verify the predictive performance of bGEBA-SVM. bGEBA-SVM was compared with eight similar methods and four popular prediction models, including bBA-SVM [22], bSCA-SVM [24], bGWO-SVM [50], bHHO-SVM [51], bSMA-SVM [21], Back Propagation Neuron NetWok (BP) [52], Classification And Regression Tree (CART) [53], Random Forest (RandomF) [54] and Adaptive Boosting (AdaBoost) [55] for comparison. To ensure the fairness of the experiment, this study unified the experimental setup. The population size (*N*) was set to 20, the dimension was determined by the dataset, and the number of loop terminations (*Max*_*Iter*) was set to 100. In addition, in order to avoid the occurrence of randomized experimental results, this study set up 10 independently run experiments and discussed the results of the mean and standard deviation.

In addition, to obtain a more accurate assessment of the model performance, this study comprehensively evaluated the model prediction ability by analyzing the four-assessment metrics of Accuracy, Sensitivity, Matthews correlation coefficient (MCC), and F-measure of the prediction results, the details of which are shown in Table 10.

**Table 10:** Evaluation metrics for forecasting methods.

Metrics	Calculation formula
**Accuracy**	$\mathrm{Accuracy}=\frac{\mathrm{TP}+\mathrm{TN}}{\mathrm{TP}+\mathrm{FP}+\mathrm{FN}+\mathrm{TN}}$
**Sensitivity**	$\mathrm{Sensitivity}=\frac{\mathrm{TP}}{\mathrm{TP}+\mathrm{FN}}$
**MCC**	$\mathrm{MCC}=\frac{\mathrm{TP}\times \mathrm{TN}‐\mathrm{FP}\times \mathrm{FN}}{\sqrt{\left(\mathrm{TP}+\mathrm{FP})\times (\mathrm{TP}+\mathrm{FN})\times (\mathrm{TN}+\mathrm{FP})\times (\mathrm{TN}+\mathrm{FN}\right)}}$
**F-measure**	$F‐\mathrm{measure}=\frac{\mathrm{TP}}{\mathrm{TP}+\frac{\mathrm{FN}+\mathrm{FP}}{2}}$

### 4.2 Transformation function experiment

When optimization methods are discretized, the use of different transformation functions can cause differences in prediction results [56]. Therefore, to improve the prediction performance of the model for the GEP dataset, this subsection conducted experimental tests for the eight most common transformation functions [57] for S-shaped and V-shaped, and the experimental setup is shown in Table 11.

**Table 11:** Details of S-shaped and V-shaped functions.

Name	Optimizer	Transfer function	Classifier
**S1-bGEBA-SVM**	GEBA	$s=\frac{1}{1+e^{-2x}}$	SVM
**S2-bGEBA-SVM**		$s=\frac{1}{1+e^{-x}}$	
**S3-bGEBA-SVM**		$s=\frac{1}{1+e^{\left(-\frac{x}{2}\right)}}$	
**S4-bGEBA-SVM**		$s=\frac{1}{1+e^{\left(-\frac{x}{3}\right)}}$	
**V1-bGEBA-SVM**		$s=\left|\frac{\sqrt{2}}{\pi }\int_{0}^{(\sqrt{\pi }/2)x}e^{-t^{2}}dt\right|$	
**V2-bGEBA-SVM**		s=|tanh(x)|	
**V3-bGEBA-SVM**		$s=\left|(x)/\sqrt{1+x^{2}}\right|$	
**V4-bGEBA-SVM**		$s=\left|\frac{2}{\pi }arctan(\frac{\pi }{2}x)\right|$	

**Fig 7:**
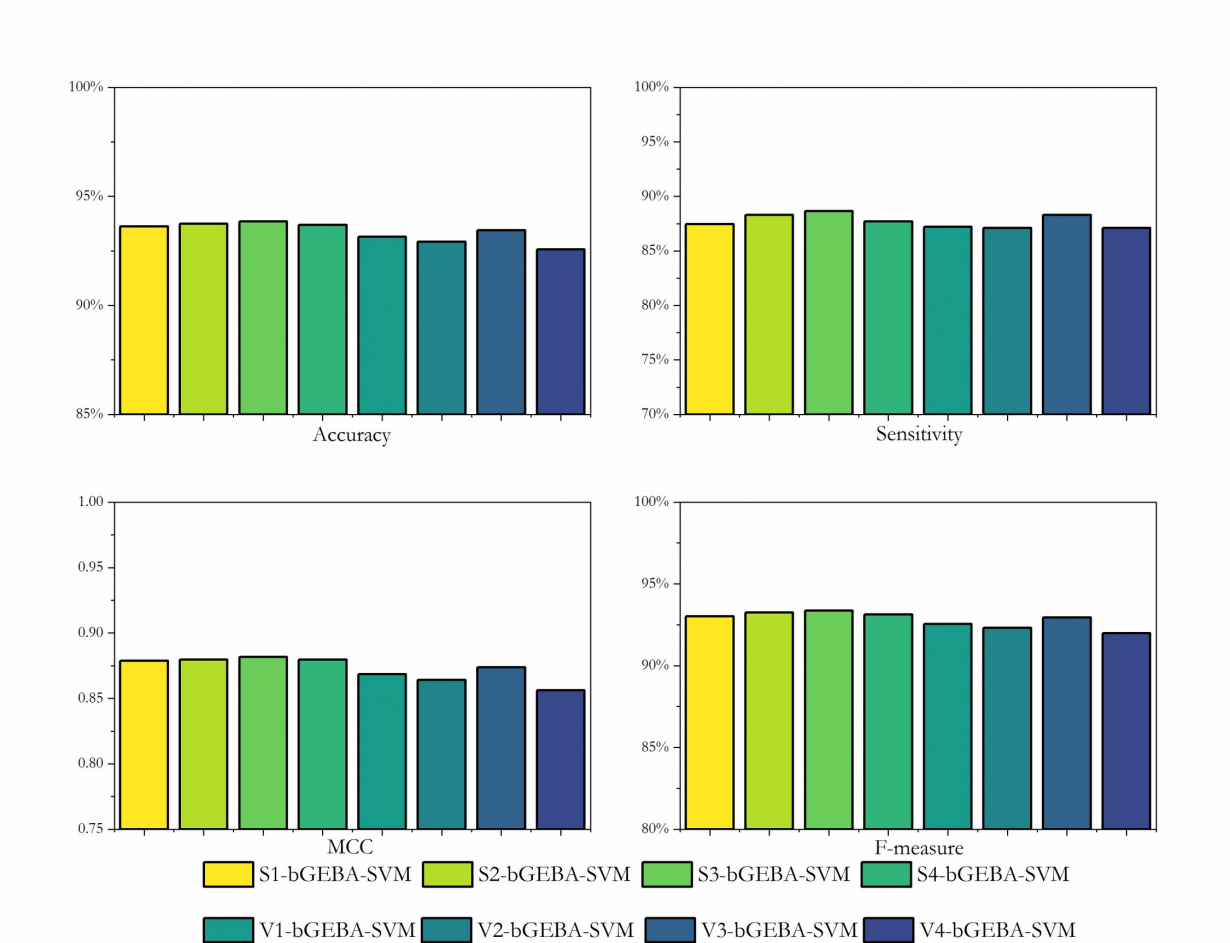


Fig 7 shows the four evaluation metrics for the prediction of the GEP dataset by eight discretization methods. As can be seen from the figure, S3-bGEBA-SVM performs the best among the eight comparisons, with Accuracy, Sensitivity, Matthews correlation coefficient, and F-measure reaching 93.86%, 88.65%, 0.8816 and 93.36%, respectively. This suggests that in order to optimize the feature space and eventually produce better prediction results for GEBA, the S3 transformation function is more appropriate. Therefore, this study set the S3 function as the default discretization method and conducted subsequent experiments.

### 4.3 Comparative experiment on the public datasets

To verify the generalization ability of the proposed method, bGEBA-SVM was compared with other methods by six public datasets [58,59] for comparative experiments, and Table 12 shows the details of these datasets.

**Table 12:** Details of the public dataset.

Datasets	Instances	Features
**Heartandlung**	139	23
**Hepatitis**	155	19
**Ionosphere**	351	34
**Vote**	300	16
**Heart**	270	13
**WDBC**	569	30

Table 13 to Table 18 shows the means and standard deviations of the four evaluation metrics for the prediction results of the public dataset for the 10 prediction methods. Observing the data of the table species, we can see that bGEBA-SVM has the best mean and standard deviation of various metrics on all datasets except for the standard deviation of the four evaluation metrics on the Heart dataset. It indicates that bGEBA-SVM has better prediction ability for public datasets and its prediction performance is stable, which is a method with excellent generalization ability.

**Table 13:** Prediction evaluation results for public dataset Heartandlung.

Algorithms	Accuracy		Sensitivity		MCC		F-measure	
	Mean	Stdv	Mean	Stdv	Mean	Stdv	Mean	Stdv
**bGEBA-SVM**	**99.29%**	2.26%	**100.00%**	0.00%	**0.9866**	0.0424	**99.33%**	2.11%
**bBA-SVM**	97.14%	3.69%	97.14%	6.02%	0.9464	0.0692	97.13%	3.72%
**bSCA-SVM**	97.09%	5.05%	95.71%	9.64%	0.9465	0.0916	96.90%	5.63%
**bGWO-SVM**	95.66%	3.74%	98.57%	4.52%	0.9184	0.0703	95.90%	3.54%
**bHHO-SVM**	97.09%	3.76%	98.57%	4.52%	0.9455	0.0704	97.23%	3.59%
**bSMA-SVM**	97.86%	4.82%	98.57%	4.52%	0.9580	0.0954	97.91%	4.77%
**BP**	91.43%	12.95%	92.86%	10.10%	0.8359	0.2563	92.03%	11.30%
**CART**	89.95%	9.63%	90.00%	9.64%	0.8024	0.1927	90.08%	9.45%
**RandomF**	89.95%	7.66%	90.00%	13.55%	0.8149	0.1354	89.78%	8.34%
**AdaBoost**	92.80%	8.91%	92.86%	12.14%	0.8629	0.1757	92.71%	9.16%

**Table 14:** Prediction evaluation results for public dataset Hepatitisfulldata.

Algorithms	Accuracy		Sensitivity		MCC		F-measure	
	Mean	Stdv	Mean	Stdv	Mean	Stdv	Mean	Stdv
bGEBA-SVM	100.00%	0.00%	100.00%	0.00%	1.0000	0.0000	100.00%	0.00%
**bBA-SVM**	99.41%	1.86%	97.50%	7.91%	0.9835	0.0523	98.57%	4.52%
**bSCA-SVM**	98.67%	2.81%	93.33%	14.06%	0.9569	0.0909	96.00%	8.43%
**bGWO-SVM**	99.38%	1.98%	96.67%	10.54%	0.9787	0.0674	98.00%	6.32%
**bHHO-SVM**	98.71%	2.72%	94.17%	12.45%	0.9617	0.0816	96.57%	7.35%
**bSMA-SVM**	98.75%	2.64%	96.67%	10.54%	0.9619	0.0811	96.57%	7.35%
**BP**	88.38%	6.77%	78.33%	26.99%	0.6857	0.1979	72.21%	18.11%
**CART**	80.08%	11.06%	45.83%	28.12%	0.4078	0.2953	48.52%	26.61%
**RandomF**	84.50%	6.79%	62.50%	25.23%	0.5442	0.2138	61.21%	18.11%
**AdaBoost**	81.36%	7.66%	40.00%	35.53%	0.3406	0.3195	40.38%	33.60%

**Table 15:** Prediction evaluation results for public dataset IonosphereEW.

Algorithms	Accuracy		Sensitivity		MCC		F-measure	
	Mean	Stdv	Mean	Stdv	Mean	Stdv	Mean	Stdv
**bGEBA-SVM**	**100.00%**	0.00%	**100.00%**	0.00%	**1.0000**	0.0000	**100.00%**	0.00%
**bBA-SVM**	99.71%	0.90%	**100.00%**	0.00%	0.9937	0.0198	99.79%	0.67%
**bSCA-SVM**	99.72%	0.88%	**100.00%**	0.00%	0.9941	0.0188	99.79%	0.67%
**bGWO-SVM**	99.71%	0.93%	**100.00%**	0.00%	0.9936	0.0201	99.78%	0.70%
**bHHO-SVM**	99.71%	0.90%	**100.00%**	0.00%	0.9940	0.0191	99.78%	0.70%
**bSMA-SVM**	99.71%	0.90%	**100.00%**	0.00%	0.9937	0.0198	99.79%	0.67%
**BP**	67.78%	17.21%	59.86%	24.20%	0.4129	0.2968	68.14%	20.34%
**CART**	94.29%	3.04%	96.46%	4.06%	0.8782	0.0663	95.58%	2.33%
**RandomF**	93.97%	5.23%	97.33%	3.75%	0.8706	0.1153	95.48%	3.89%
**AdaBoost**	92.63%	3.80%	97.33%	3.14%	0.8410	0.0827	94.43%	2.91%

**Table 16:** Prediction evaluation results for public dataset Vote.

Algorithms	Accuracy		Sensitivity		MCC		F-measure	
	Mean	Stdv	Mean	Stdv	Mean	Stdv	Mean	Stdv
**bGEBA-SVM**	**99.67%**	1.05%	**100.00%**	0.00%	**0.9932**	0.0215	**99.57%**	1.37%
**bBA-SVM**	99.34%	1.38%	**100.00%**	0.00%	0.9869	0.0277	99.20%	1.69%
**bSCA-SVM**	99.00%	2.25%	99.17%	2.64%	0.9802	0.0442	98.73%	2.83%
**bGWO-SVM**	98.99%	1.63%	99.09%	2.87%	0.9794	0.0333	98.69%	2.12%
**bHHO-SVM**	99.33%	1.41%	99.17%	2.64%	0.9866	0.0283	99.17%	1.76%
**bSMA-SVM**	99.32%	1.43%	99.09%	2.87%	0.9862	0.0292	99.12%	1.86%
**BP**	84.67%	5.94%	84.70%	12.97%	0.6989	0.1220	80.99%	6.78%
**CART**	95.34%	3.92%	96.52%	6.22%	0.9060	0.0793	94.16%	4.93%
**RandomF**	94.74%	4.85%	95.00%	7.03%	0.8923	0.1002	93.35%	6.03%
**AdaBoost**	95.64%	3.21%	94.77%	6.22%	0.9110	0.0661	94.35%	4.16%

**Table 17:** Prediction evaluation results for public dataset Heart.

Algorithms	Accuracy		Sensitivity		MCC		F-measure	
	Mean	Stdv	Mean	Stdv	Mean	Stdv	Mean	Stdv
**bGEBA-SVM**	**95.19%**	3.51%	**98.00%**	4.50%	**0.9052**	0.0694	**95.76%**	3.19%
**bBA-SVM**	94.82%	3.12%	96.00%	4.66%	0.8983	0.0620	95.37%	2.82%
**bSCA-SVM**	94.82%	3.58%	96.67%	3.51%	0.8965	0.0723	95.42%	3.14%
**bGWO-SVM**	94.82%	3.58%	97.33%	4.66%	0.9000	0.0681	95.47%	3.05%
**bHHO-SVM**	94.44%	4.00%	95.33%	4.50%	0.8887	0.0807	95.01%	3.61%
**bSMA-SVM**	94.82%	3.98%	96.67%	4.71%	0.8987	0.0771	95.43%	3.46%
**BP**	78.15%	5.94%	72.67%	12.97%	0.5659	0.1220	78.57%	6.78%
**CART**	76.30%	3.92%	80.67%	6.22%	0.5300	0.0793	79.13%	4.93%
**RandomF**	79.26%	4.85%	83.33%	7.03%	0.5862	0.1002	81.62%	6.03%
**AdaBoost**	81.85%	3.21%	86.00%	6.22%	0.6357	0.0661	84.13%	4.16%

**Table 18:** Prediction evaluation results for public dataset wdbc.

Algorithms	Accuracy		Sensitivity		MCC		F-measure	
	Mean	Stdv	Mean	Stdv	Mean	Stdv	Mean	Stdv
**bGEBA-SVM**	**99.83%**	0.55%	**100.00%**	0.00%	**0.9964**	0.0113	**99.78%**	0.70%
**bBA-SVM**	99.65%	0.75%	99.05%	2.01%	0.9925	0.0158	99.51%	1.03%
**bSCA-SVM**	99.47%	0.85%	98.57%	2.30%	0.9888	0.0181	99.27%	1.18%
**bGWO-SVM**	99.65%	0.74%	99.05%	2.01%	0.9925	0.0158	99.51%	1.03%
**bHHO-SVM**	99.48%	0.84%	98.62%	2.23%	0.9890	0.0178	99.29%	1.14%
**bSMA-SVM**	99.47%	0.85%	99.05%	2.01%	0.9889	0.0179	99.28%	1.16%
**BP**	80.65%	8.81%	79.59%	10.16%	0.6096	0.2111	75.18%	8.63%
**CART**	92.26%	7.03%	91.54%	9.66%	0.8383	0.1529	89.81%	5.48%
**RandomF**	96.30%	6.81%	94.35%	9.56%	0.9216	0.1375	94.97%	6.30%
**AdaBoost**	97.37%	7.70%	94.35%	7.34%	0.9444	0.1564	96.33%	6.47%

### 4.4 Comparative experiment on the employment prediction dataset

In this subsection, to achieve a more accurate prediction of graduate employment, bGEBA-SVM was used to predict the GEP dataset, and its superior prediction performance was verified by comparing it with other methods.

**Fig 8:**
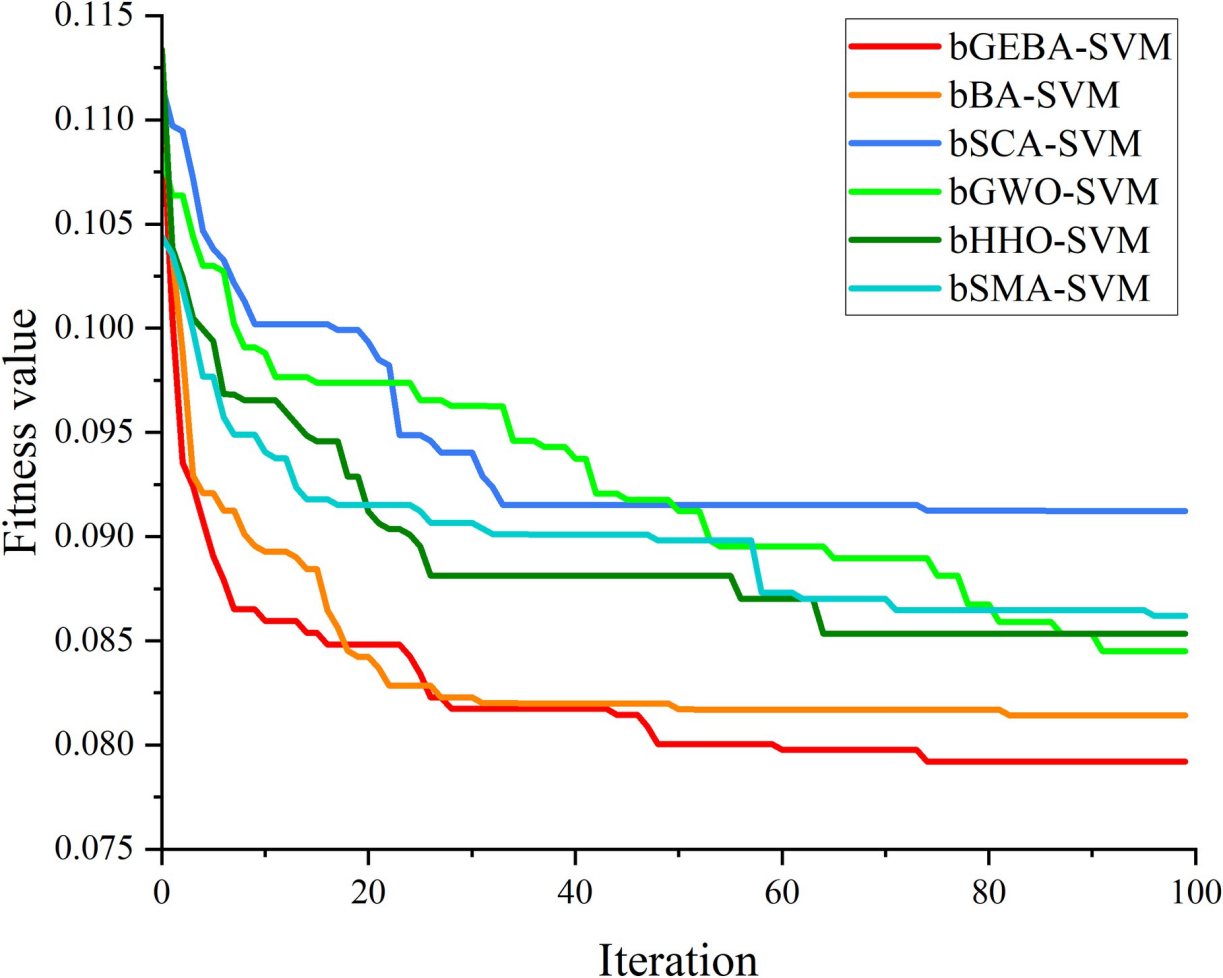


**Fig 9:**
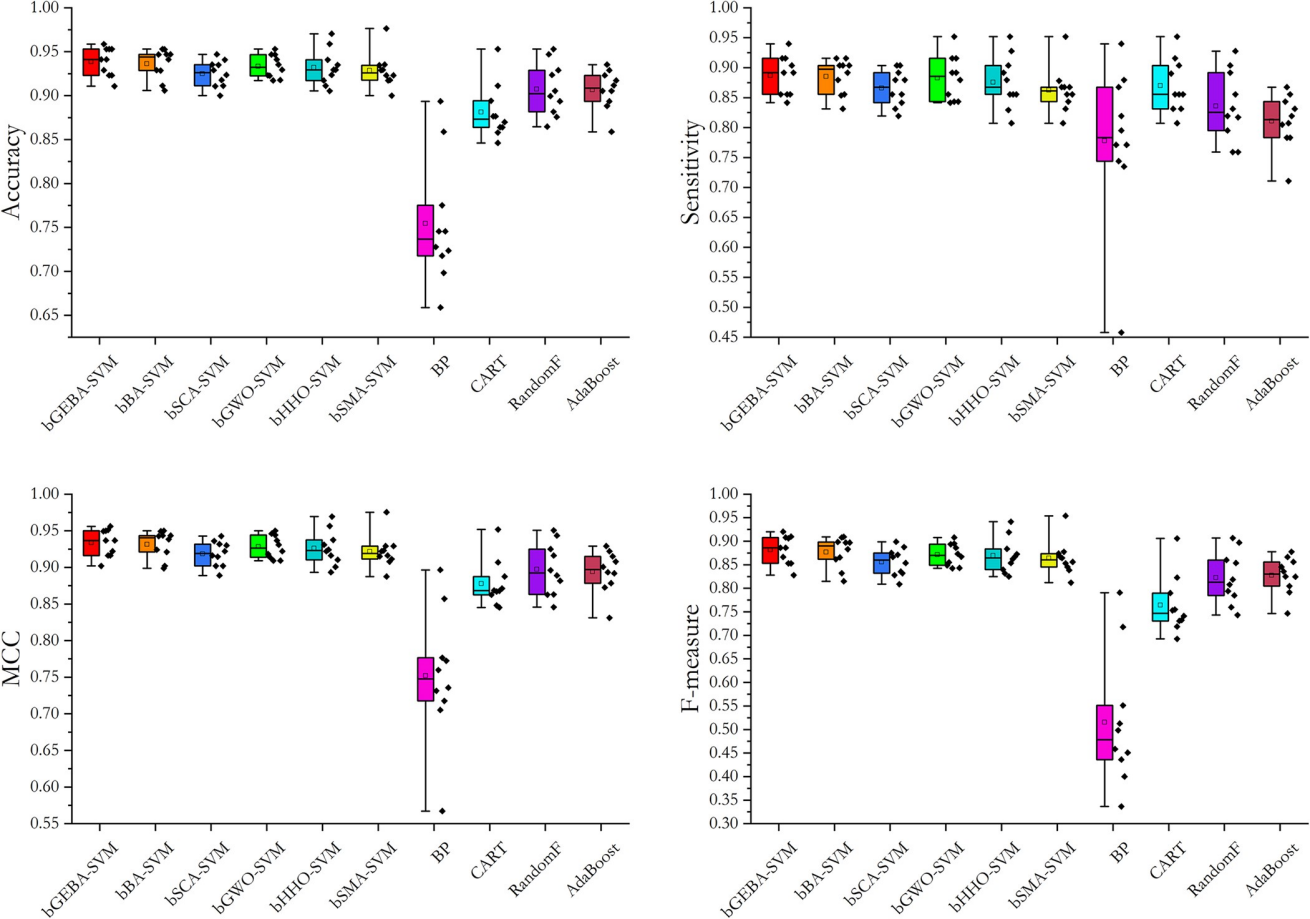


In the wrapper feature selection method, bGEBA was used as an optimization method to optimize a subset of features, and the set of features that favor the prediction accuracy of the model was fed into the classifier. Among them, the optimization performance of bGEBA is a key factor affecting the prediction accuracy of the model. Therefore, the convergence curves of bGEBA as well as peers for evaluating the objective function of feature subsets were analyzed as shown in Fig 8. Observing the images, it can be seen that bGEBA can jump out of the local optimum compared with other methods, and thus obtain a higher-quality feature subset. Combining the optimization results of bGEBA for feature subsets and the above prediction results, it is clear that bGEBA has a stronger global optimization ability and further improves the prediction accuracy of the model by feeding high-quality training data to the model. Table 19 and Fig 9 show the prediction results for the graduate employment dataset. It is easy to see that bGEBA-SVM can achieve a more accurate prediction of graduate employment due to peers and popular prediction models on the four performance evaluation metrics.

**Table 19:** Prediction evaluation results for the employment prediction dataset.

Algorithms	Accuracy		Sensitivity		MCC		F-measure	
	Mean	Stdv	Mean	Stdv	Mean	Stdv	Mean	Stdv
**bGEBA-SVM**	**93.86%**	1.63%	**88.66%**	3.30%	**0.8816**	0.0305	**93.36%**	1.85%
**bBA-SVM**	93.62%	1.69%	88.54%	2.93%	0.8766	0.0326	93.13%	1.89%
**bSCA-SVM**	92.50%	1.50%	86.61%	2.99%	0.8557	0.0290	91.86%	1.72%
**bGWO-SVM**	93.33%	1.31%	88.29%	3.75%	0.8712	0.0236	92.80%	1.56%
**bHHO-SVM**	93.21%	2.04%	87.57%	4.42%	0.8696	0.0372	92.61%	2.37%
**bSMA-SVM**	92.86%	1.97%	86.25%	3.77%	0.8641	0.0369	92.16%	2.24%
**BP**	75.45%	7.16%	77.80%	13.00%	0.5152	0.1404	75.20%	8.90%
**CART**	88.13%	3.12%	86.98%	4.46%	0.7642	0.0617	87.77%	3.15%
**RandomF**	90.73%	3.00%	83.59%	5.83%	0.8225	0.0555	89.73%	3.57%
**AdaBoost**	90.68%	2.24%	81.06%	4.51%	0.8274	0.0389	89.42%	2.86%

### 4.5 Important features analysis

The proposed bGEBA-SVM method has strong interpretability, and to verify the role played by each feature in the prediction process, feature importance experiments were set up in this study.

**Fig 10:**
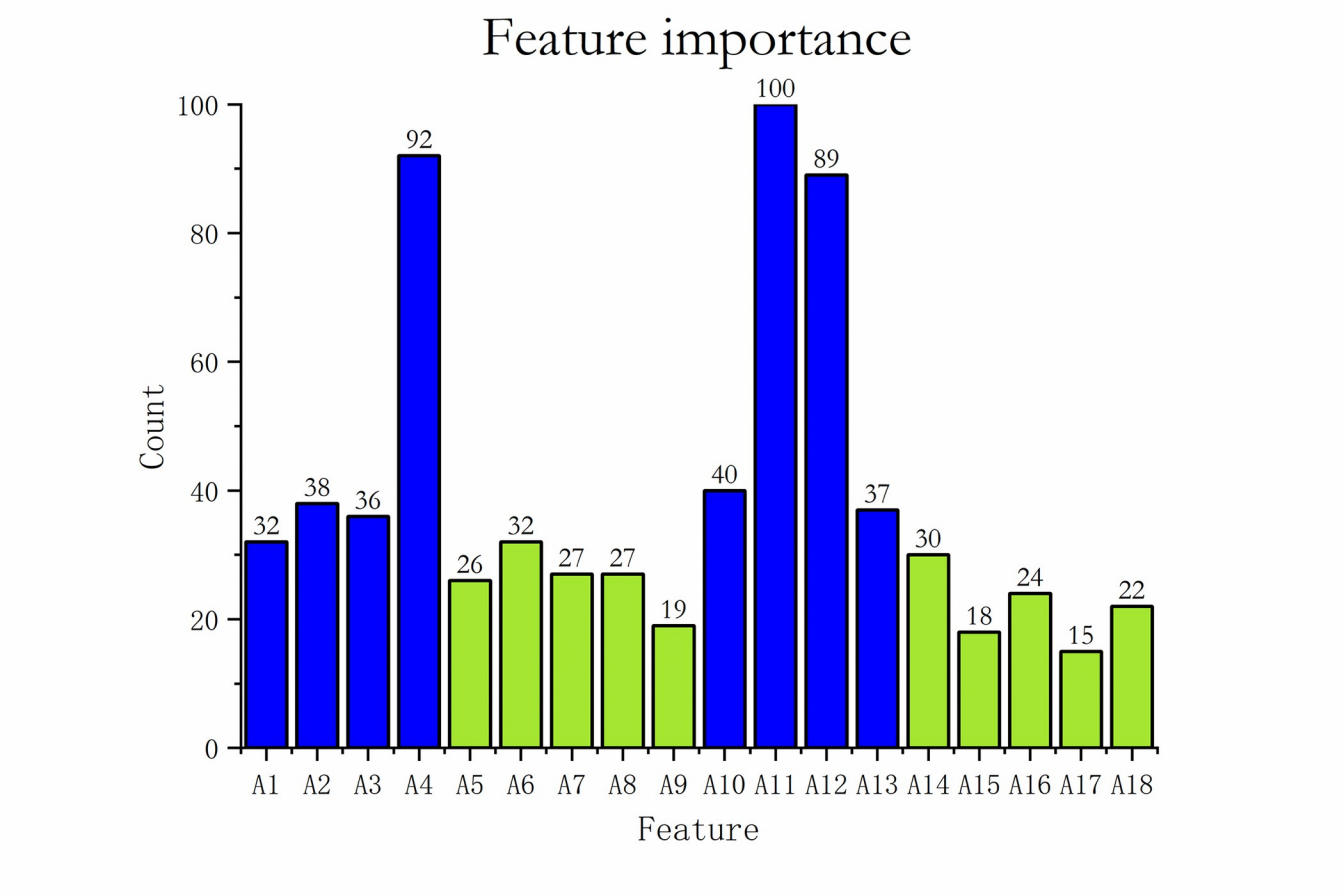


Fig 10 shows the number of times each feature was selected in the 100 times of feature selection experiments. The experimental results are realistic in that the features A11, A4, A12, A10, A2, A13, A3, and A1 are selected more times and have a more significant impact on predicting the employment aspect of graduates.

## 5 Discussion

The results of the experiment show that there are significant correlations among the factors influencing college students’ choice of ’slow employment’, such as preparation for school, student leadership experience, family situation, career planning, employment structure, family parenting style, student category, gender, professional interest. Among them, career planning, academic achievement, self-concept, and family situation are negatively correlated with the intention of ’slow employment’, i.e. the clearer the career planning, the better the academic achievement, the clearer the self-concept and the better the family economic situation, the lower the intention of ’slow employment’ of college students [60,61]. There are three other aspects that we should consider. First, among the ’slow employment’ intention, the number of students who are actively ’slow employment’ is increasing because they are pursuing further education, and more of them have the experience of being student leaders during their college years. Among those who are actively ’slow employment’, there is one point worth noting: there are more science and technology majors than economics and management majors, while agriculture majors have a lower intention to be actively ’slow employment’ [61–63]. Second, family upbringing also influences students’ intention of ’slow employment’, the more democratic family upbringing, the stronger students’ intention of ’slow employment’. Excessive family interference or lack of family involvement can make career decision-making difficult, leading young people to make poor choices based on these interferences [2]. Relatively democratic families, on the other hand, allow young people to be less influenced by external pressures and more diversified in their career choices, but this is more likely to lead to missed opportunities. Family influence is certainly a factor worth noting in the case of slow employment. Third, students’ intention to ’slow employment’ is influenced by the type of birth source to a certain extent, and students from rural areas have a higher intention to ’slow employment’ than those from urban areas. It can also be concluded that the situation of ’slow employment’ of college students should be analyzed scientifically and treated rationally.

This paper established a scientific ’slow employment’ prediction and evaluation model, used the improved bat algorithm as a feature subset search method, and used the screened key features for the training of the classification model, the proposed evaluation model can provide reasonable auxiliary decision-making suggestions for universities to solve the problem of ’slow employment’ of college students. First, it can help colleges and universities to do a good job of classifying and guiding students’ employment, predicting employment intentions, grasping the employment needs and difficulties of ’slow employment’ students in time, and providing employment guidance and services for different types of students. Second, it can help colleges and universities to make precise policies, find out the bottom number, send jobs, target help, and implement personalized employment services. Third, it can help all parties to help promote student employment. Colleges and universities can work with families, society, and other parties to help graduates understand the employment situation, strengthen their ability to improve, master the pace of job hunting, better intervene in the ’slow employment’ behavior, and actively guide their positive transformation. We can assess, predict and prevent ’slow employment’, to promote higher quality and fuller employment of graduates.

## 6 Conclusion and future works

The wrapper feature selection approach (bGEBA-SVM) presented in this study was developed on an improved bat algorithm and a support vector machine. By incorporating an Elimination approach and a Gaussian distribution-based approach into the Bat algorithm, an effective and reliable optimization technique (GEBA) was presented. In order to ensure that better feature subsets can be obtained, this study introduced the Gaussian distribution-based strategy and the Elimination strategy based on the original bat algorithm. The experimental findings at IEEE CEC2017 shown that bGEBA offers considerable optimization performance benefits over advanced comparable algorithms. bGEBA was objectively evaluated with certain sophisticated approaches. To suggest a discrete version for feature selection, the GEBA is further discretized (bGEBA). bGEBA provides effective training data for support vector machines and achieves more accurate prediction of graduate employment prediction dataset. Through experimental tests and analysis, we found that school, student leadership experience, family situation, career planning, employment structure, family parenting style, student category, gender, professional interest and other factors have a greater influence on graduates’ positive and negative responses to the choice of "slow employment". Analyzing these factors facilitates a more accurate prediction of graduate employment problems and the development of effective measures.

Although the proposed model has a more stable and excellent performance for the graduate "slow employment" prediction problem. However, its performance is still limited by the optimization efficiency and classification model performance. In future research, we will plan to incorporate high-performance computing techniques such as distributed optimization to solve the feature subset optimization efficiency problem, and use machine learning models with stronger predictive performance and compatibility. Additionally, the suggested GEBA’s optimization capabilities may be utilized to address picture segmentation [64] and engineering optimization challenges [65].
